# MyD88 and IL-1R signaling drive antibacterial immunity and osteoclast-driven bone loss during *Staphylococcus aureus* osteomyelitis

**DOI:** 10.1371/journal.ppat.1007744

**Published:** 2019-04-12

**Authors:** Nicole E. Putnam, Laura E. Fulbright, Jacob M. Curry, Caleb A. Ford, Jenna R. Petronglo, Andrew S. Hendrix, James E. Cassat

**Affiliations:** 1 Department of Pathology, Microbiology and Immunology, Vanderbilt University Medical Center, Nashville, Tennessee, United States of America; 2 Department of Pediatrics, Division of Pediatric Infectious Diseases, Vanderbilt University Medical Center, Nashville, Tennessee, United States of America; 3 Department of Biomedical Engineering, Vanderbilt University, Nashville, Tennessee, United States of America; 4 Vanderbilt Institute for Infection, Immunology and Inflammation (VI4), Vanderbilt University Medical Center, Nashville, Tennessee, United States of America; 5 Vanderbilt Center for Bone Biology, Vanderbilt University Medical Center, Nashville, Tennessee, United States of America; Johns Hopkins School of Medicine, UNITED STATES

## Abstract

*Staphylococcus aureus* is able to infect virtually all organ systems and is a frequently isolated etiologic agent of osteomyelitis, a common and debilitating invasive infection of bone. Treatment of osteomyelitis requires invasive surgical procedures and prolonged antibiotic therapy, yet is frequently unsuccessful due to extensive pathogen-induced bone damage that can limit antibiotic penetration and immune cell influx to the infectious focus. We previously established that *S*. *aureus* triggers profound alterations in bone remodeling in a murine model of osteomyelitis, in part through the production of osteolytic toxins. However, staphylococcal strains lacking osteolytic toxins still incite significant bone destruction, suggesting that host immune responses are also major drivers of pathologic bone remodeling during osteomyelitis. The objective of this study was to identify host immune pathways that contribute to antibacterial immunity during *S*. *aureus* osteomyelitis, and to define how these immune responses alter bone homeostasis and contribute to bone destruction. We specifically focused on the interleukin-1 receptor (IL-1R) and downstream adapter protein MyD88 given the prominent role of this signaling pathway in both antibacterial immunity and osteo-immunologic crosstalk. We discovered that while IL-1R signaling is necessary for local control of bacterial replication during osteomyelitis, it also contributes to bone loss during infection. Mechanistically, we demonstrate that *S*. *aureus* enhances osteoclastogenesis of myeloid precursors *in vitro*, and increases the abundance of osteoclasts residing on bone surfaces *in vivo*. This enhanced osteoclast abundance translates to trabecular bone loss, and is dependent on intact IL-1R signaling. Collectively, these data define IL-1R signaling as a critical component of the host response to *S*. *aureus* osteomyelitis, but also demonstrate that IL-1R-dependent immune responses trigger collateral bone damage through activation of osteoclast-mediated bone resorption.

## Author summary

Osteomyelitis is a common, debilitating infection of bone that rarely resolves without prolonged antibiotics and invasive surgical procedures. This study explores the role of host inflammation during osteomyelitis. Our findings highlight innate immune responses that are critical for control of *S*. *aureus* burdens in bone, prevention of bacterial dissemination, and death. Conversely, these same immune pathways were found to contribute to disease pathogenesis by activating infection-associated bone loss. Such bone loss is associated with detrimental outcomes during osteomyelitis, including pathologic fractures and irreversible changes in bone growth plates. Alterations in bone remodeling that occur during *S*. *aureus* osteomyelitis are multifaceted, driven by bacterial toxins and inflammation-mediated changes. We found that inflammation incited by *S*. *aureus* in bone contributes to pathologic bone loss. We highlight a mechanism by which *S*. *aureus* stimulates formation of bone-resorbing osteoclasts both *in vitro* and *in vivo*. Our data illustrate connections between innate immune signaling pathways and bone homeostasis. These connections may be relevant to other autoinflammatory bone and joint conditions characterized by bone loss, such as rheumatoid arthritis. Collectively, this work outlines the fine balance between promotion of bacterial clearance and protection from collateral tissue damage.

## Introduction

Osteomyelitis, or inflammation of bone, is most commonly caused by invasive bacterial infection [1]. *S*. *aureus* is the most frequently isolated etiologic agent of both acute and chronic bacterial osteomyelitis [2, 3]. *S*. *aureus* can colonize bone through hematogenous dissemination, contamination of bone following surgical or accidental trauma, or direct spread from a surrounding soft tissue infection [2, 4]. Bone represents a unique niche for invading bacterial pathogens as it is constantly undergoing turnover by bone-forming osteoblasts and bone-resorbing osteoclasts. Bone also represents a distinctive immunological niche, as bone marrow houses hematopoietic stem cells that give rise to lymphocytes and myeloid cells [5]. Bone infections rarely resolve without medical intervention, and are difficult to treat due to the widespread antimicrobial resistance of *S*. *aureus* as well as induction of bone damage that effectively limits antibiotic delivery and immune cell influx [2, 3].

Osteomyelitis elicits pathologic bone remodeling, which in addition to contributing to treatment failure, can enhance the likelihood of complications such as pathologic fractures [2, 6–11]. In order to explore mechanisms of bone loss during osteomyelitis, we previously established a murine model of post-traumatic osteomyelitis [6]. Using this model, we identified a subset of staphylococcal toxins, the alpha-type phenol soluble modulins (PSMs), that are responsible for killing primary bone cells *in vitro* and that enhance bone destruction *in vivo* [6, 7]. However, a *S*. *aureus* strain lacking the alpha-type PSMs still incites substantial bone damage, causing approximately 70% of the bone loss that is observed in femurs infected with a wild-type (WT) *S*. *aureus* strain [6]. PSMα toxins, along with many other staphylococcal toxins and proteases, are regulated by the *agr* quorum sensing system. In rabbit and murine models of experimental osteomyelitis, inactivation of *agr* further reduced bone destruction [6, 11]. However, significant cortical bone loss still occurred even with this virulence-attenuated strain in our model of post-traumatic osteomyelitis [6]. Taken together, these findings indicate that while bacterial factors directly contribute to bone damage, a substantial proportion of bone loss during osteomyelitis may be caused by host factors [12].

In order to maintain skeletal strength and structure, bone must be continuously remodeled by bone-forming osteoblasts and bone-resorbing osteoclasts [13, 14]. In addition to a major role in bone formation, osteoblast lineage cells are the major cellular regulators of bone-resorption through the balanced production of a TNF-family cytokine known as receptor activator of NFκB ligand (RANKL), and the RANKL decoy molecule osteoprotegerin (OPG). RANKL production favors bone resorption by acting on osteoclasts, which are multinucleated bone-degrading cells that differentiate from the myeloid lineage. Bone remodeling occurs as a part of normal vertebrate physiology, but the kinetics of bone remodeling can be substantially altered in response to local and systemic inflammation [15]. Osteomyelitis, in particular, is associated with abundant levels of pro-inflammatory cytokines such as TNFα, IL-1β, and IL-6 [5, 16]. These pro-inflammatory cytokines have been shown to promote skeletal cell differentiation *in vitro*, both directly by stimulating bone-resorbing osteoclasts and indirectly by promoting osteoblast production of RANKL to drive osteoclastogenesis [15, 17, 18]. IL-1 in particular was formerly referred to as “osteoclast activating factor,” reflecting the ability of IL-1α and IL-1β to signal on osteoclast lineage cells to increase osteoclast viability and resorptive capacity [19–24]. Through these mechanisms, pro-inflammatory cytokines contribute to bone loss *in vivo* in non-infectious models of rheumatoid arthritis [17, 25, 26], although less is known about their influence on bone loss during osteomyelitis. These observations led us to hypothesize that *S*. *aureus* osteomyelitis triggers enhanced bone loss through pro-inflammatory cytokine production and signaling.

IL-1 cytokines signal downstream of the IL-1R through the adapter protein MyD88, which also transduces signals from various Toll-like receptors (TLRs) after ligation by conserved microbial motifs known as pathogen-associated molecular patterns (PAMPs). Thus, MyD88 is a critical component of the innate immune system, by relaying signals through the IL-1R and many TLRs. Prior research has highlighted a prominent role for MyD88 and IL-1R signaling in the activation of immune responses that are necessary to control *S*. *aureus* infection in other animal models of infection [27–35]. In part, this occurs through the ability of IL-1 to mediate neutrophil recruitment and promote proper abscess formation for containment of *S*. *aureus* [27, 30]. Moreover, IL-1 plays a critical role in potentiating granulopoiesis, which occurs primarily in the bone marrow [36, 37]. The expansion and recruitment of granulocytes, such as neutrophils, are regulated in part by an IL-1R-dependent mechanism by which IL-1 signals onto endothelial cells in the bone marrow to release G-CSF [38–40]. Thus, MyD88 and the IL-1R form a critical signaling cascade that is necessary to mount an effective immune response to invading pathogens.

Importantly, osteoblasts and osteoclasts express innate immune receptors through which these cells sense and respond to PAMPs and inflammatory cytokines in cell culture [26]. Given the important role of IL-1 in anti-staphylococcal immunity, as well as compelling evidence demonstrating that IL-1 signaling impacts bone cells *in vitro*, we hypothesized that MyD88 and IL-1R signaling are required for efficient antibacterial immune responses during osteomyelitis, but paradoxically may also promote pathologic bone loss. To test this hypothesis, we used a murine model of *S*. *aureus* osteomyelitis, high resolution imaging, histologic analyses, and *in vitro* skeletal cell assays. We show that IL-1 is abundantly produced in bone in response to *S*. *aureus* infection, and that MyD88 and IL-1R signaling are required to limit staphylococcal burdens during osteomyelitis. Furthermore, *S*. *aureus* incites bone loss *in vivo* through an IL-1R-mediated increase in osteoclastogenesis. Our findings reveal that while MyD88 and IL-1R signaling are necessary for antibacterial responses in bone, they also contribute to *S*. *aureus*-stimulated osteoclastogenesis and host-mediated bone loss during osteomyelitis.

## Results

### *S*. *aureus* osteomyelitis alters cortical and trabecular bone remodeling

**Fig 1:**
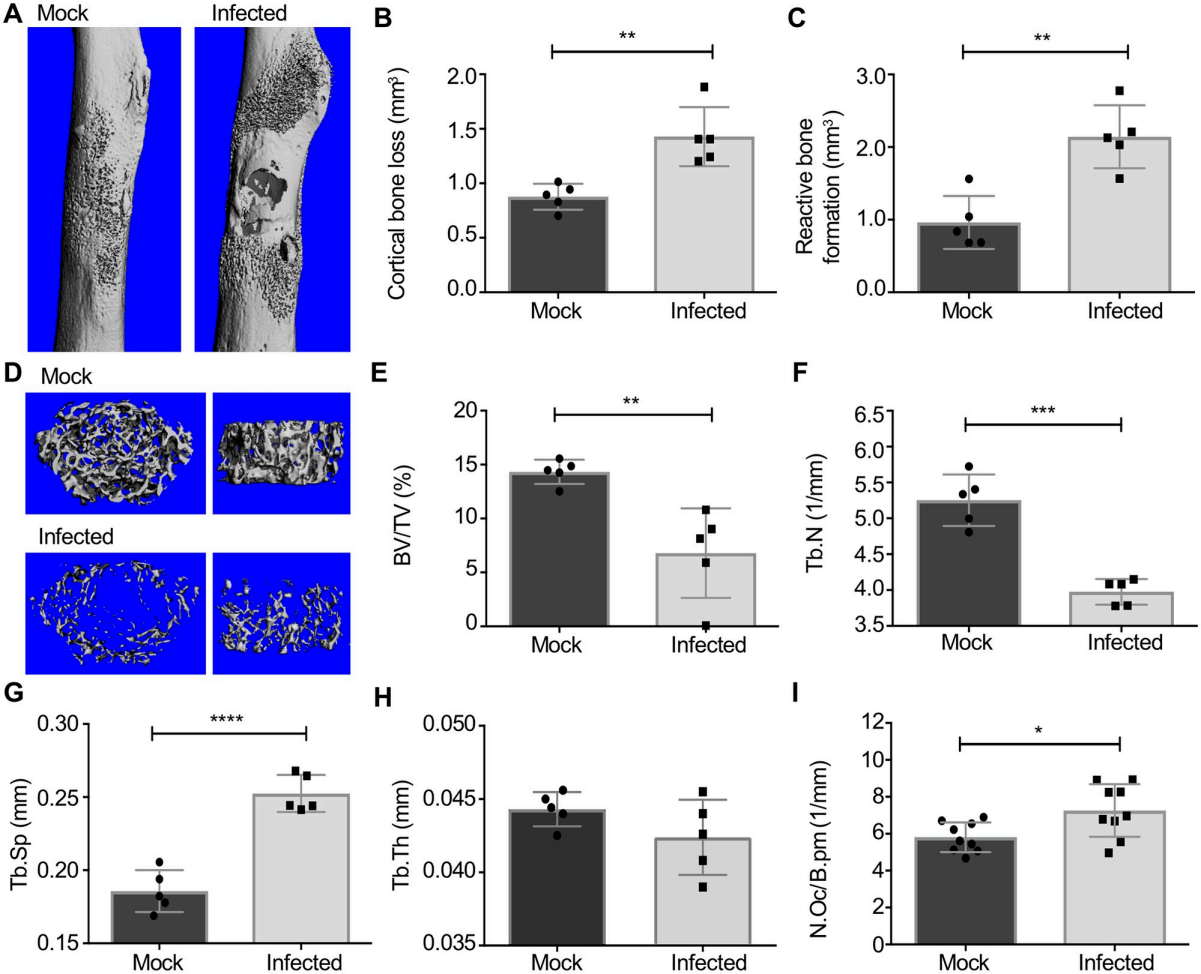
*S. aureus* osteomyelitis alters cortical and trabecular bone remodeling. (A-H) Mock infection or osteomyelitis was induced in female wild-type C57BL/6J (WT) mice via intraosseous inoculation with PBS or *S. aureus*. At 14 days post-infection, femurs were harvested, fixed in neutral buffered formalin, and scanned with a μCT50 at 10 μm resolution (*n* = 5 mice per group). (A) Anteroposterior view of the femur at the inoculation site in representative mock infected and *S. aureus* infected femurs. (B, C) Cortical bone loss (mm^3^) (B) and reactive bone formation (mm^3^) (C) were quantified via μCT. (D) Representative three-dimensional top down (left) and side (right) views of trabecular bone architecture in mock infected and *S. aureus* infected femurs. (E-H) Trabecular bone indices, including three-dimensional measurements of trabecular bone volume/total volume (BV/TV) (%) (E), trabecular number (Tb.N) (1/mm) (F), trabecular spacing (Tb.Sp) (mm) (G), and trabecular thickness (Tb.Th) (mm) (H) were measured via μCT. After scanning, femurs were decalcified, processed, and embedded in paraffin for histologic sectioning and TRAP staining. (I) Histomorphometric analyses of trabecular bone in the distal femur proximal to the growth plate compared the number of osteoclasts per bone perimeter (N.Oc/B.pm) (1/mm) between mock infected and *S. aureus* infected femurs (*n* = 9 mice per group). Symbols represent individual data points from each mouse (Mock = circles; Infected = squares), the top line of each bar represents the mean, and error bars represent standard deviation. Unpaired *t*-tests were used to compare μCT and histomorphometry measurements between mock infected and *S. aureus* infected femurs. * *p* < 0.05, ** *p* < 0.01, *** *p* < 0.001, **** *p* < 0.0001.

In order to determine changes in bone remodeling that occur during osteomyelitis, we compared architectural bone parameters between infected and mock infected wild-type (WT) C57BL/6J mice in a post-traumatic model of *S*. *aureus* bone infection [6]. We focused our analyses on two distinct anatomical sites of the infected femurs representing the two major architectural types of bone: cortical bone that comprises the mid-region (diaphysis) of the long bone and trabecular bone found in the distal femur (metaphysis and epiphysis) (S1A–S1C Fig). We previously observed that mock infected WT mice display a rapid cortical bone healing response at the surgical site, in which the induced bone defect in the femoral diaphysis is replaced with new bone by 2 weeks post-surgery [6]. In contrast to this sterile cortical bone repair, mice infected with *S*. *aureus* develop osteomyelitis, are unable to restore the cortical bone defect, and experience extensive cortical bone loss surrounding the site of inoculation (Fig 1A and 1B). Moreover, *S*. *aureus* infected femurs show reactive cortical bone formation surrounding the site of inoculation (Fig 1C) [6].

*S*. *aureus* osteomyelitis induces dramatic alterations in cortical bone surrounding the infectious focus, which was initiated in the middle of the femoral diaphysis. However, trabecular bone, located at the ends of the long bones, is the major site of homeostatic bone remodeling [41]. In order to elucidate how inflammation during osteomyelitis leads to alterations in trabecular bone architecture, we also performed micro-computed tomography (μCT) imaging on trabecular bone in the distal femur. To determine the amount of trabecular bone that was lost during osteomyelitis, we calculated the trabecular bone volume per total volume (BV/TV), which is a standard measure of bone volume and architecture [42]. *S*. *aureus* infected femurs exhibited a dramatic loss in trabecular bone, with BV/TV markedly decreased in infected femurs compared to mock infected femurs (Fig 1D and 1E). The observed decrease in BV/TV during infection is reflective of a decline in the number of bony trabeculae, which in turn increases the overall volume of space between trabeculae (Fig 1F and 1G). Trabecular thickness was not significantly reduced in infected relative to mock infected femurs (Fig 1H). Although skeletal histology revealed that the area of the femur encompassing the trabecular bone did not have apparent abscess formation (S1C Fig), viable *S*. *aureus* cells were recoverable from the femoral epiphyses encompassing the trabecular bone (S2 Fig). These data collectively reveal that *S*. *aureus* osteomyelitis induces changes in bone turnover throughout the entire infected femur, which is reflected in a significant loss of cortical and trabecular bone.

One common mechanism of bone loss is mediated by an increase in the number of bone-resorbing osteoclasts residing on the bone surface. To examine if the inflammation associated with *S*. *aureus* osteomyelitis enhances numbers of osteoclasts *in vivo*, we collected histologic sections of infected femurs for histomorphometry, which enables quantification of the number of osteoclasts and osteoclast resorbing surface relative to intact trabecular bone. Histomorphometric analysis showed an increased number of osteoclasts per bone perimeter (N.Oc/B.pm) in *S*. *aureus* infected femurs relative to mock infected femurs, suggesting that enhancement of osteoclastogenesis might be one mechanism underlying trabecular bone loss during osteomyelitis (Fig 1I). Taken together, these data indicate that *S*. *aureus* osteomyelitis perturbs normal bone homeostasis to induce pathologic bone remodeling in both cortical and trabecular bone.

### Longitudinal cytokine profiling defines the local inflammatory milieu during *S*. *aureus* osteomyelitis

**Fig 2:**
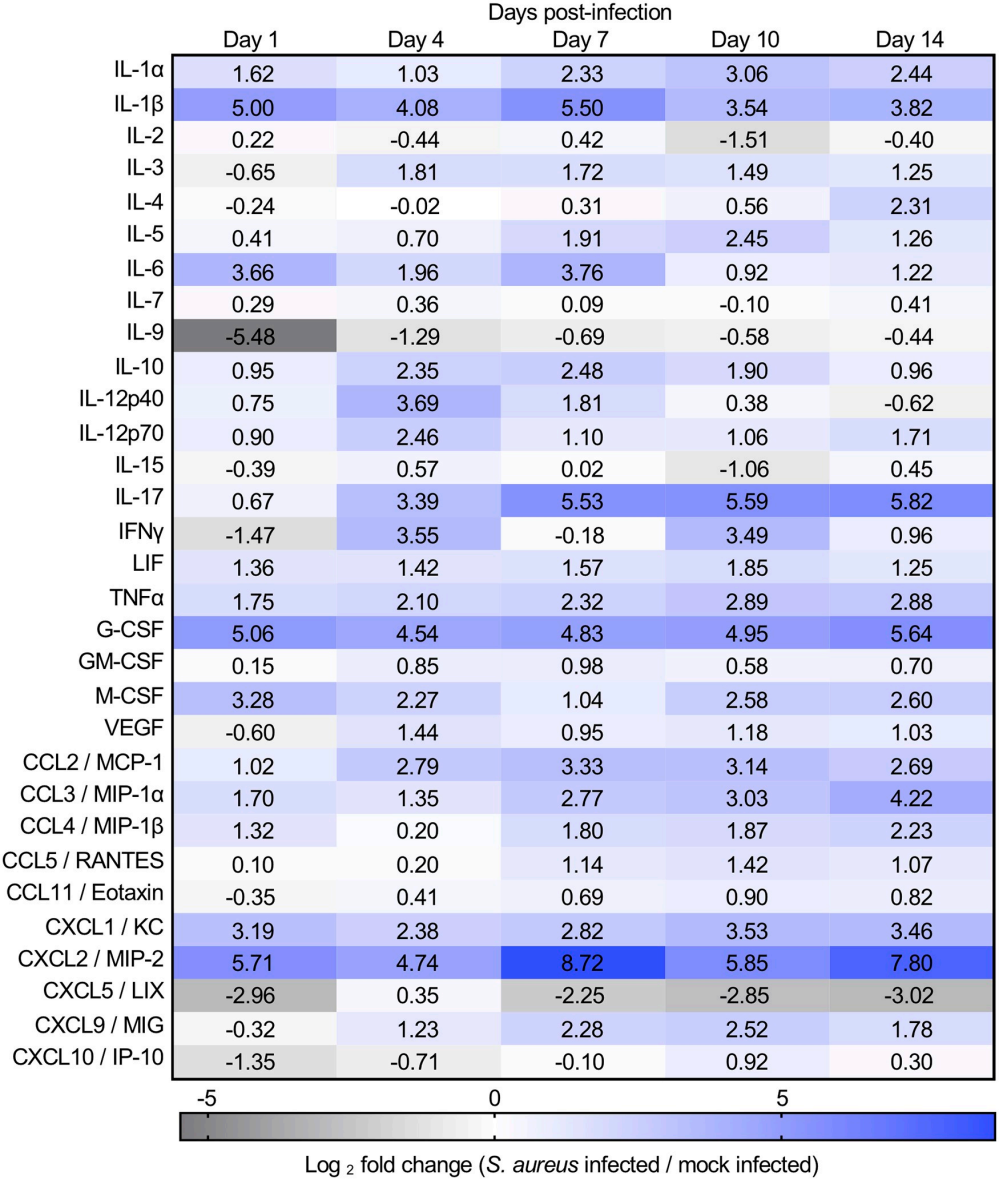
Longitudinal cytokine profiling defines the local inflammatory milieu during *S. aureus* osteomyelitis. Cytokine profiling was performed on homogenates from mock and *S. aureus* infected femurs from female WT mice. Groups of mock or *S. aureus* infected mice (*n* = 3 per time point) were sacrificed on days 1, 4, 7, 10, and 14 post-infection. Relative cytokine levels are represented as Log_2_(*S. aureus* infected/mock infected) to reflect changes in cytokine production between sterile and infected bones. Dark blue shading represents increasing cytokine fold-changes observed in *S. aureus* infected femurs compared to mock infected femurs. Dark grey shading represents a decreasing cytokine fold-changes in *S. aureus* infected femurs compared to mock infected femurs.

Previous studies have shown that toxin-deficient *S*. *aureus* strains retain the ability to alter bone remodeling, albeit to a lesser extent than WT *S*. *aureus*, implicating inflammation as a potential mediator of dysregulated bone remodeling during osteomyelitis [6, 7]. To characterize the local inflammatory environment during *S*. *aureus* osteomyelitis, we conducted longitudinal, multiplexed cytokine profiling of *S*. *aureus* and mock infected femurs over the course of 14 days. Relative to mock infected femurs, *S*. *aureus* infected femurs have more abundant levels of cardinal pro-inflammatory cytokines including IL-1α, IL-1β, IL-6, and TNFα (Fig 2). While both IL-1α and IL-1β are highly produced in infected femurs, IL-1β had a higher fold change than IL-1α throughout the timecourse when comparing *S*. *aureus* infected to mock infected femurs. Furthermore, infected femurs have increased levels of cytokines that support myeloid cell chemotaxis and expansion, including KC (CXCL1), G-CSF, M-CSF, MCP-1 (CCL2), MIP-1α (CCL3), MIP-1β (CCL4), and MIP-2 (CXCL2), compared to mock infected femurs (Fig 2). Cytokine profiling of *S*. *aureus* osteomyelitis demonstrated that inflammatory cytokines, chemokines, and growth factors are greatly increased in infected femurs by day 1 and throughout infection.

### The innate immune signaling adapter MyD88 and IL-1R signaling are critical for the control of bacterial burdens during *S*. *aureus* osteomyelitis

**Fig 3:**
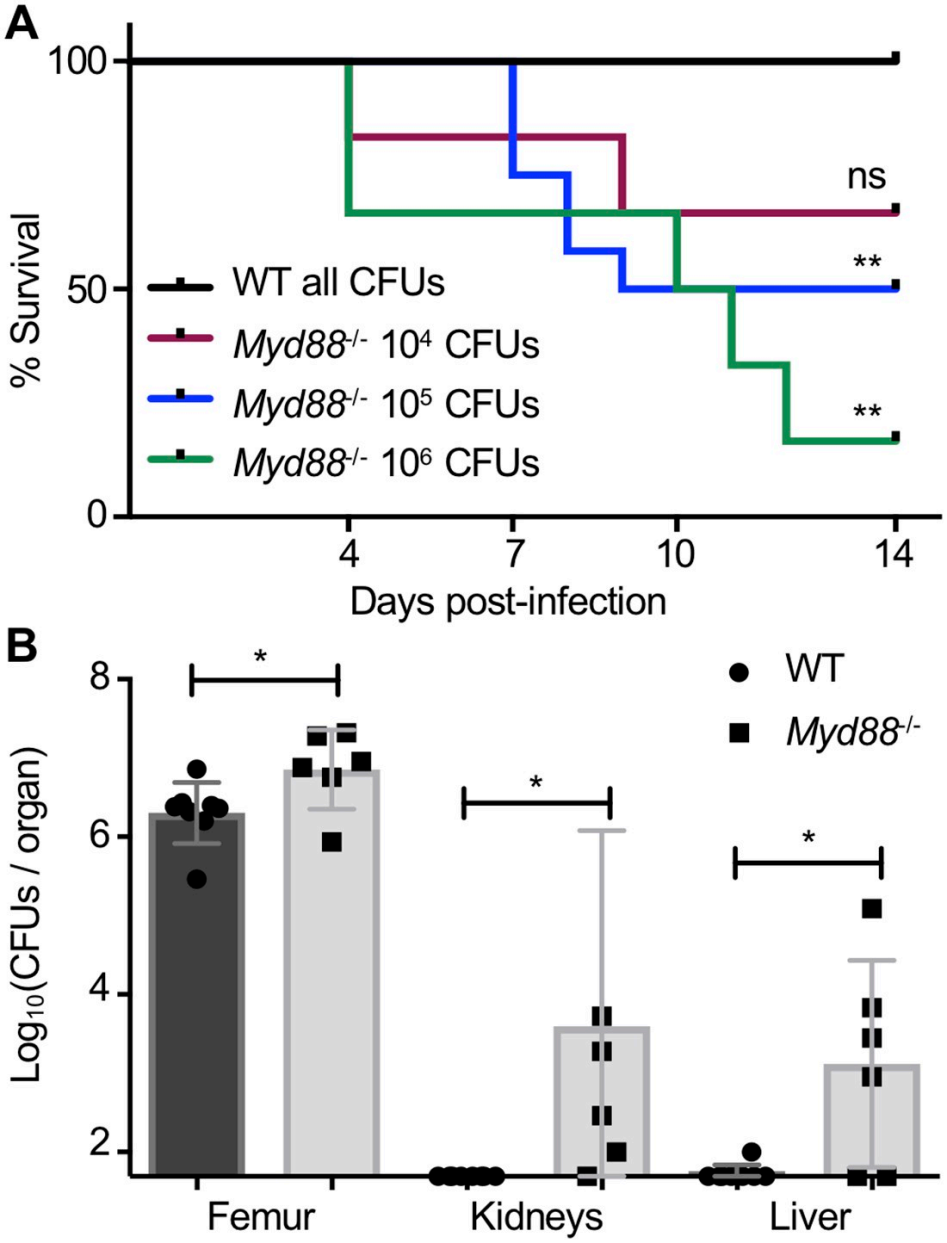
The innate immune signaling adapter MyD88 is critical for the control of bacterial burdens and systemic dissemination during *S. aureus* osteomyelitis. (A-B) Osteomyelitis was established in female WT and *Myd88*^-/-^ mice, following infection with varying *S. aureus* inocula: 10^6^ CFUs (WT *n* = 5, *Myd88*^-/-^ = 6), 10^5^ CFUs (WT *n* = 10, *Myd88*^-/-^ = 12; duplicate experiments), 10^4^ CFUs (WT *n* = 6, *Myd88*^-/-^ = 6). Bacterial burdens were quantified from the infected femur, as well as both kidneys and the liver as a measure of bacterial dissemination. (A) Over the 14-day course of infection, mice were monitored for humane endpoints, and if necessary, mice were euthanized, and mortality was recorded. Log-rank Mantel Cox test was used to compare WT and *Myd88*^-/-^ survival curves due to infection mortality for each *S. aureus* inoculum. ** *p* < 0.01, ns = not significant. (B) Bacterial burdens were enumerated in surviving mice at day 14 post-infection in duplicate experiments, following inoculation with 10^5^ *S. aureus* CFUs (WT *n* = 8, *Myd88*^-/-^ *n* = 6; duplicate experiments). Symbols represent individual data points from each mouse (WT = circles; *Myd88*^-/-^ = squares), the top line of each bar represents the mean, and error bars represent standard deviation. Unpaired *t*-tests were used to compare CFU burdens between WT and *Myd88*^-/-^ organ homogenates. * *p* < 0.05, ns = not significant.

A rapid and robust cytokine response to *S*. *aureus* in bone led us to focus on identifying host signaling pathways that are responsible for coordinating an innate immune response. Given the central role for the signaling adapter MyD88 in pathogen recognition and induction of innate immune responses, we first sought to determine how MyD88 signaling influences staphylococcal burdens and host morbidity and mortality during osteomyelitis. *Myd88*^-/-^ mice have enhanced susceptibility to, and morbidity from, bacterial infection [27, 43, 44]. We therefore inoculated these mice with a range of *S*. *aureus* colony forming units (CFUs), from 10^4^−10^6^. Although bacterial inocula up to 10^6^ CFUs did not cause mortality in WT mice, *Myd88*^-/-^ mice were exquisitely susceptible to *S*. *aureus* osteomyelitis, with mortality observed even at inocula as low as 10^4^ CFUs (Fig 3A). For infected *Myd88*^-/-^ mice that met humane endpoints prior to the experimental endpoint or succumbing to disease, bacterial CFUs were enumerated at that time in the femur, liver, and kidneys. At the time of early sacrifice, *Myd88*^-/-^ mice had between 10^7^−10^8^ *S*. *aureus* CFUs in the femur, kidneys, and liver, which was significantly increased over CFUs recovered from WT mice (S3 Fig). At the experimental endpoint (day 14 post-infection), surviving *Myd88*^-/-^ mice not only had significantly elevated bacterial burdens in the infected femur, but also experienced more bacterial dissemination to the kidneys and liver (Fig 3B). Consequently, the inability to prevent systemic bacterial dissemination results in significantly increased mortality in *Myd88*^-/-^ mice. Taken together, these data demonstrate a critical role for MyD88-dependent immune responses during *S*. *aureus* osteomyelitis.

*Myd88*^*-/-*^ mice have altered intestinal barrier function and are severely immunocompromised, and therefore may have significant microbiome differences relative to WT mice [45–47]. When considered in concert with recent studies suggesting that the microbiome may regulate bone mass [48–51], these observations prompted us to breed *Myd88*^*-/-*^ and *Myd88*^*+/+*^ littermate controls from a heterozygous colony and compare these littermate controls for susceptibility to osteomyelitis. We also examined the influence of sex as a biologic variable in these experiments. In line with results from mice bred in separate colonies, significant mortality from osteomyelitis was observed in male *Myd88*^*-/-*^ mice, but not *Myd88*^*+/+*^ littermate controls (S4A Fig). Of note, at day 14 post-infection, male *Myd88*^*-/-*^ mice had no difference from *Myd88*^*+/+*^ littermate controls in recovered CFUs from infected femurs (S4B Fig). This observation could indicate sex-dependent differences in osteomyelitis pathogenesis, or alternatively may reflect selection bias from removal of mice that succumbed to infection (S4A Fig). In contrast to male mice, female *Myd88*^*-/-*^ mice exhibited significantly higher bacterial burdens in the infected femur when compared *Myd88*^*+/+*^ littermate controls (S4C Fig). These experiments confirm that MyD88 is critical for the control of bacterial burdens and systemic dissemination during osteomyelitis independently of any confounding variables associated with separate colony maintenance.

**Fig 4:**
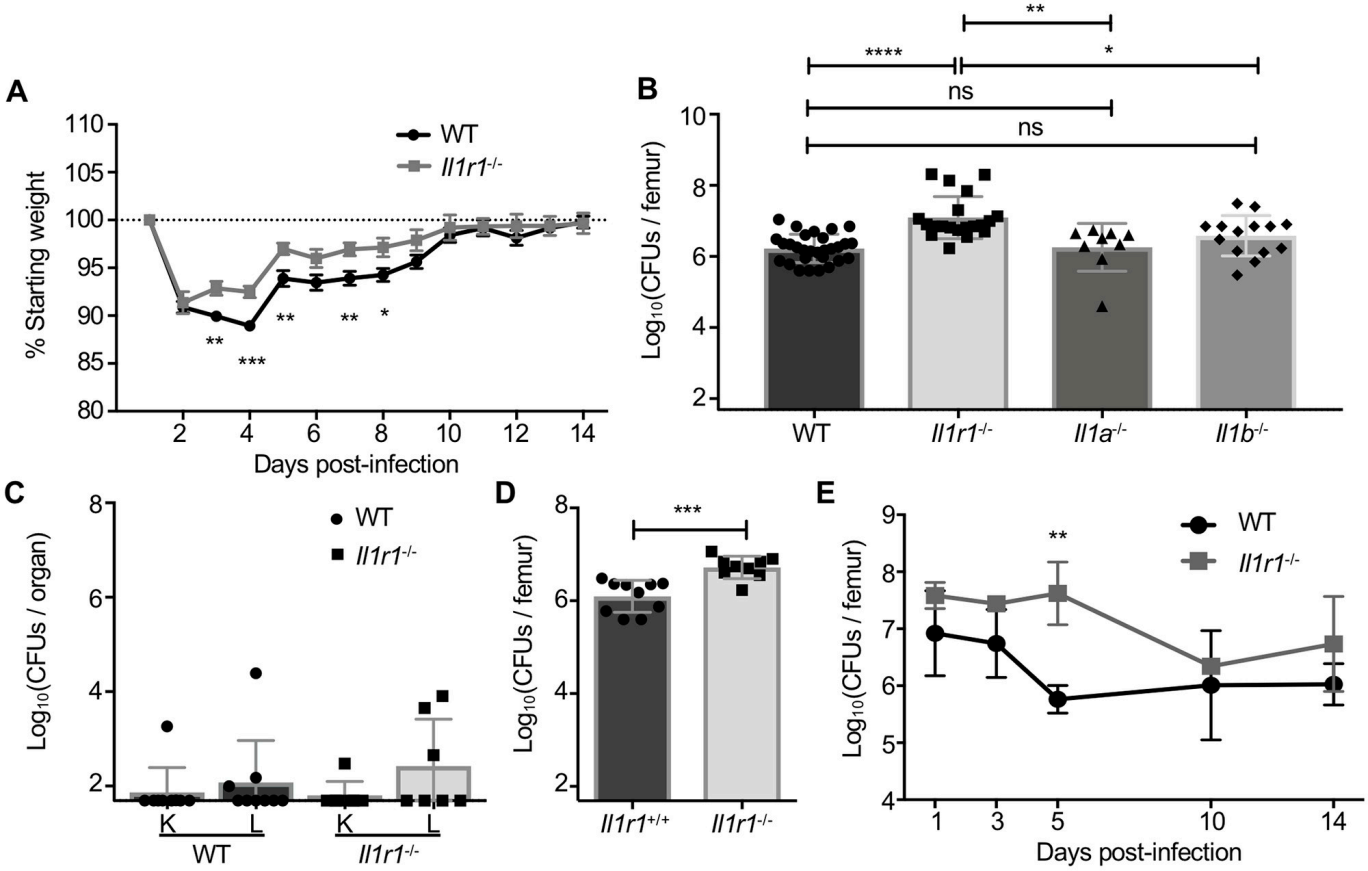
IL-1R signaling contributes to antibacterial immunity in bone. (A) Percent starting weight was monitored daily in triplicate experiments with 10^6^ *S. aureus* CFU infection of female WT and *Il1r1*^-/-^ mice (WT *n* = 15, *Il1r1*^-/-^ *n* = 13). (B-C) At day 14 post-infection with 10^6^ *S. aureus* CFU, bacterial burdens were enumerated from the infected femurs (WT *n* = 29, *Il1r1*^-/-^ *n* = 20, *Il1a*^-/-^ *n* = 9, *Il1b*^-/-^ *n* = 14; six independent experiments) (B) or kidneys (K) and liver (L) (WT *n* = 9, *Il1r1*^-/-^ *n* = 7; duplicate experiments) (C) of each mouse. (D) A subsequent experiment with a 10^6^ *S. aureus* CFU inoculum was conducted in *Il1r1*^+/+^ and *ll1r1*^-/-^ mice bred as littermates from a heterozygous colony (*Il1r1*^+/+^ *n* = 10, *ll1r1*^-/-^ *n* = 9; duplicate experiments). (E) Following a lower 10^5^ *S. aureus* CFU inoculum, bacterial burdens were enumerated from infected femurs of WT and *Il1r1*^-/-^ mice on days 1, 3, 5, 10, and 14 post-infection (*n* = 3 mice per genotype per time point). On bar graphs, symbols represent individual data points from a single mouse (WT and *Il1r1*^+/+^ = circles; *Il1r1*^-/-^ = squares; *Il1a*^-/-^ = triangles, *Il1b*^-/-^ = diamonds) and the mean is represented by the top line of the bar. On timecourse graphs, symbols represent the mean. On all graphs, error bars represent the standard deviation. Unpaired *t*-tests compared weight recovery and CFU burdens between WT and *Il1r1*^-/-^ mice and between *Il1r1*^+/+^ and *Il1r1*^-/-^ femurs. A one-way ANOVA with Tukey’s multiple comparisons test was used to compare bacterial burdens harvested from femurs between mice of all genotypes. For kidney and liver burdens, separate *t*-tests compared WT and *Il1r1*^-/-^ burdens from each organ site. * *p* < 0.05, ** *p* < 0.01, *** *p* < 0.001, **** *p* < 0.0001, ns = not significant.

In the absence of MyD88 signaling, mice are unable to control *S*. *aureus* infection, indicating that upstream receptors that signal through MyD88, including *S*. *aureus*-recognizing TLRs and IL-1R, may be important for antibacterial protection. The high levels of IL-1 and IL-1-regulated cytokines present in *S*. *aureus* infected femurs led us to investigate the contribution of IL-1R signaling to anti-staphylococcal immunity in bone. We subjected WT and *Il1r1*^*-/-*^ mice to *S*. *aureus* osteomyelitis. In contrast to the extreme systemic morbidity observed in *MyD88*^-/-^ mice suffering from osteomyelitis, *Il1r1*^*-/-*^ mice had less morbidity when compared to WT mice, in that they lost significantly less weight over the course of infection (Fig 4A). To determine the role of IL-1R and the relative contributions of IL-1 isoforms (IL-1α or IL-1β) to control bacterial burdens in bone, WT, *Il1r1*^-/-^, *Il1a*^-/-^, and *Il1b*^-/-^ mice were subjected to *S*. *aureus* osteomyelitis. Enumeration of bacterial burdens revealed that *Il1r1*^*-/-*^ mice harbored significantly higher bacterial burdens in infected femurs than WT, *Il1a*^-/-^, and *Il1b*^-/-^ mice (Fig 4B). *Il1a*^-/-^ and *Il1b*^-/-^ mice sustained bacterial burdens that were not significantly different from WT mice (Fig 4B). Unlike *MyD88*^-/-^ mice, *Il1r1*^-/-^ mice were protected from significant systemic dissemination to the liver or kidneys (Fig 4C). To determine whether differences in WT and *Il1r1*^-/-^ strains were due to background genotype or separate colony maintenance, heterozygous *Il1r1*^*+/-*^ mice were bred to generate *Il1r1*^*+/+*^ and *Il1r1*^*-/-*^ littermate controls. Infection of littermates with 10^6^ *S*. *aureus* CFUs confirmed that *Il1r1*^*-/-*^ mice sustained higher bacterial burdens in bone compared to *Il1r1*^*+/+*^ mice (Fig 4D).

We next investigated the kinetics of bacterial clearance between WT and *Il1r1*^*-/-*^ mice. For this analysis we chose a lower *S*. *aureus* inoculum of 10^5^ CFUs in an attempt to equilibrate bacterial burdens at day 14 post-infection. In both WT and *Il1r1*^*-/-*^ mice, the initial *S*. *aureus* inoculum of 10^5^ CFUs replicates to approximately 10^7^ CFUs by day 1 post-infection (Fig 4E). In WT mice, bacterial burdens decreased by greater than 1 log between days 3 and 5 post-infection. In contrast, bacterial burdens in *Il1r1*^-/-^ mice were essentially unchanged through day 5 post-infection, and only declined between days 5 and 10 post-infection. Accordingly, WT and *Il1r1*^-/-^ mice had significantly different bacterial burdens at day 5 post-infection with this lower inoculum, even though bacterial burdens were roughly equivalent at the final time point (day 14). These data reveal differences in infection kinetics between WT and *Il1r1*^*-/-*^ mice, and suggest that *Il1r1*^-/-^ mice might have a delay in bacterial control during osteomyelitis.

**Fig 5:**
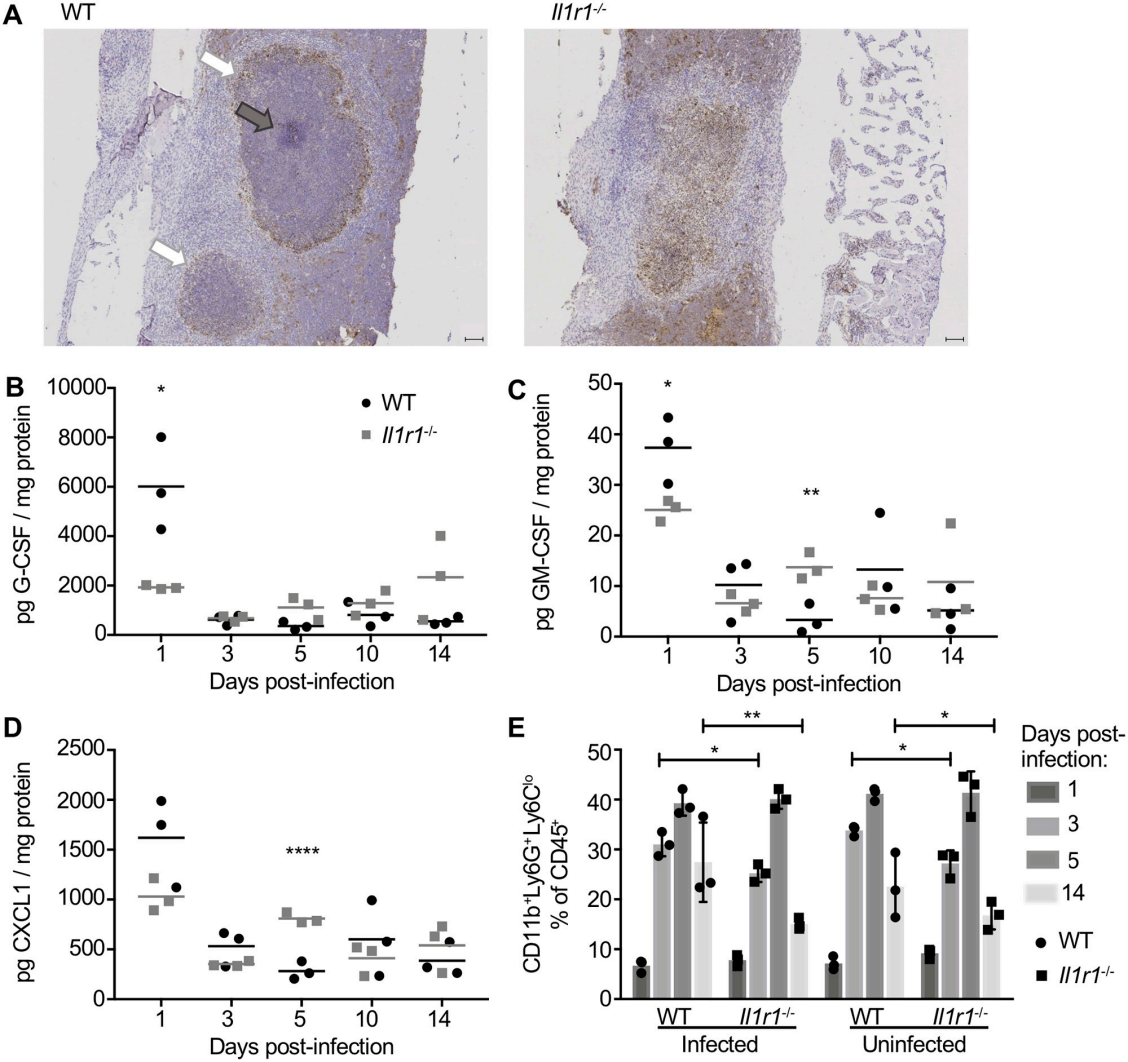
*Il1r1*^-/-^ mice have altered abscess structure, delayed granulocytic cytokine levels, and lower neutrophil abundance during *S. aureus* osteomyelitis. (A) Femurs were harvested from female WT and *Il1r1*^-/-^ mice (*n* = 5 per genotype) 14 days after *S. aureus* infection (10^6^ CFUs). Harvested femurs were fixed in neutral buffered formalin, dehydrated in 70% ethanol, and decalcified in 20% EDTA before embedding in paraffin for sectioning. The infected femurs were sectioned through the medullary cavity and sections from each femur were stained for myeloperoxidase (MPO) to visualize MPO^+^ (brown) neutrophils and gross abscess architecture in WT (left) and *Il1r1*^-/-^ (right) sections. Slides were scanned at 20X using a Leica SCN400 Slide Scanner and images were taken at 4X (scale bars = 100 μm) and are representative of *n* = 5 mice per genotype. Abscesses are indicated by white arrows and a *S. aureus* microcolony by a grey arrow. (B-D) After infection with 10^5^ *S. aureus* CFUs, cytokine levels and relative neutrophil abundance were measured from femurs (*n* = 3 per genotype) harvested at days 1, 3, 5, 10, and 14 post-infection (cytokine analysis, B-D) or days 1, 3, 5, and 14 post-infection (flow cytometry, E). Cytokine levels in pg/mL were corrected for overall protein levels in the femur homogenates as measured using a BCA assay. Depicted are select cytokines G-CSF (B), GM-CSF (C), and CXCL1 (D) from WT (black) and *Il1r1*^-/-^ (grey) femurs. (E) Relative neutrophil abundance was measured in bone marrow harvested from infected and contralateral, uninfected femurs of WT and *Il1r1*^-/-^ mice. Neutrophils are defined as CD45^+^CD11b^+^Ly6G^+^Ly6C^lo^ and are reported as % of CD45^+^ immune cells (D). Symbols represent individual data points from each mouse (WT = circles; *Il1r1*^-/-^ = squares), horizontal lines or the top of each bar represent the mean, and error bars represent standard deviation. Multiple unpaired *t*-tests were used to compared pg cytokine/mg protein and neutrophil percentages between WT and *Il1r1*^-/-^ mice. * *p* < 0.05, ** *p* < 0.01, **** *p* < 0.0001. If not denoted with asterisks, statistical difference between genotypes was not significant.

In other *S*. *aureus* infection models, IL-1 coordinates neutrophil recruitment and is necessary for sequestration of *S*. *aureus* into mature abscesses [27, 30]. We therefore hypothesized that the delay in bacterial clearance in *Il1r1*^-/-^ mice subjected to osteomyelitis was related to differences in abscess maturation and neutrophil abundance. To visualize immune cell infiltration and abscess structure, we conducted myeloperoxidase (MPO) staining on histologic sections of infected femurs at day 14 post-infection. *Il1r1*^*-/-*^ mice with osteomyelitis have differential MPO staining in comparison to WT controls, suggesting these mice have disorganized abscess structure (Fig 5A). WT mice have MPO^+^ cells that surround and encompass the abscess, whereas *Il1r1*^*-/-*^ mice show extensive MPO^+^ staining throughout the femur. To assess changes in inflammatory signatures that correspond to differences in infection kinetics, infected femur homogenates from WT and *Il1r1*^*-/-*^ mice were analyzed using multiplexed cytokine analysis. In comparison to WT mice, *Il1r1*^*-/-*^ mice had significantly decreased abundance of neutrophil growth factors G-CSF and GM-CSF and lower levels of the neutrophil chemokine CXCL1 at day 1 post-infection, a timepoint that precedes early bacterial control in WT mice between days 3 and 5 (Figs 5B–5D and 4E). GM-CSF and CXCL1 levels then decline in WT mice by day 5 post-infection. In contrast, *Il1r1*^*-/-*^ mice display significantly higher levels of GM-CSF and CXCL1 at day 5 post-infection when compared to WT mice, prior to the decrease in bacterial burdens that occurs between days 5 and 10 post-infection (Figs 5C, 5D and 4E). Moreover, there are global changes in cytokine abundance when comparing WT and *Il1r1*^-/-^ mice (S1 Table). These data suggest that WT mice have an early influx and/or expansion of neutrophils, important for the control of bacterial burdens and normal abscess formation.

To monitor neutrophil abundance during the course of osteomyelitis, bone marrow from infected and contralateral, uninfected WT and *Il1r1*^-/-^ femurs at various time points after *S*. *aureus* infection was analyzed via flow cytometry (S5A–S5F Fig). Neutrophils were identified as CD45^+^CD11b^+^Ly6G^+^Ly6C^lo^ and reported as the percent of CD45^+^ immune cells. At day 1 post-infection, WT and *Il1r1*^-/-^ mice were found to have neutrophils comprising less than 10% of CD45^+^ cells in the bone marrow. By day 3 post-infection, *Il1r1*^-/-^ mice have significantly fewer neutrophils in the infected bone marrow compared to WT mice (Fig 5E). Furthermore, differences between relative neutrophil abundance were also observed between WT and *Il1r1*^-/-^ mice in the contralateral, uninfected femurs. Neutrophil abundance at day 5 post-infection is comparable between WT and *Il1r1*^-/-^ genotypes, but again was significantly decreased in *Il1r1*^-/-^ femurs at day 14 post-infection. Therefore, the data suggest that *Il1r1*^-/-^ mice with *S*. *aureus* osteomyelitis have altered neutrophil responses, indicated by the significant decrease in relative neutrophil abundance at two timepoints post-infection.

### *S*. *aureus* promotes osteoclastogenesis and pathologic bone loss through IL-1R signaling

**Fig 6:**
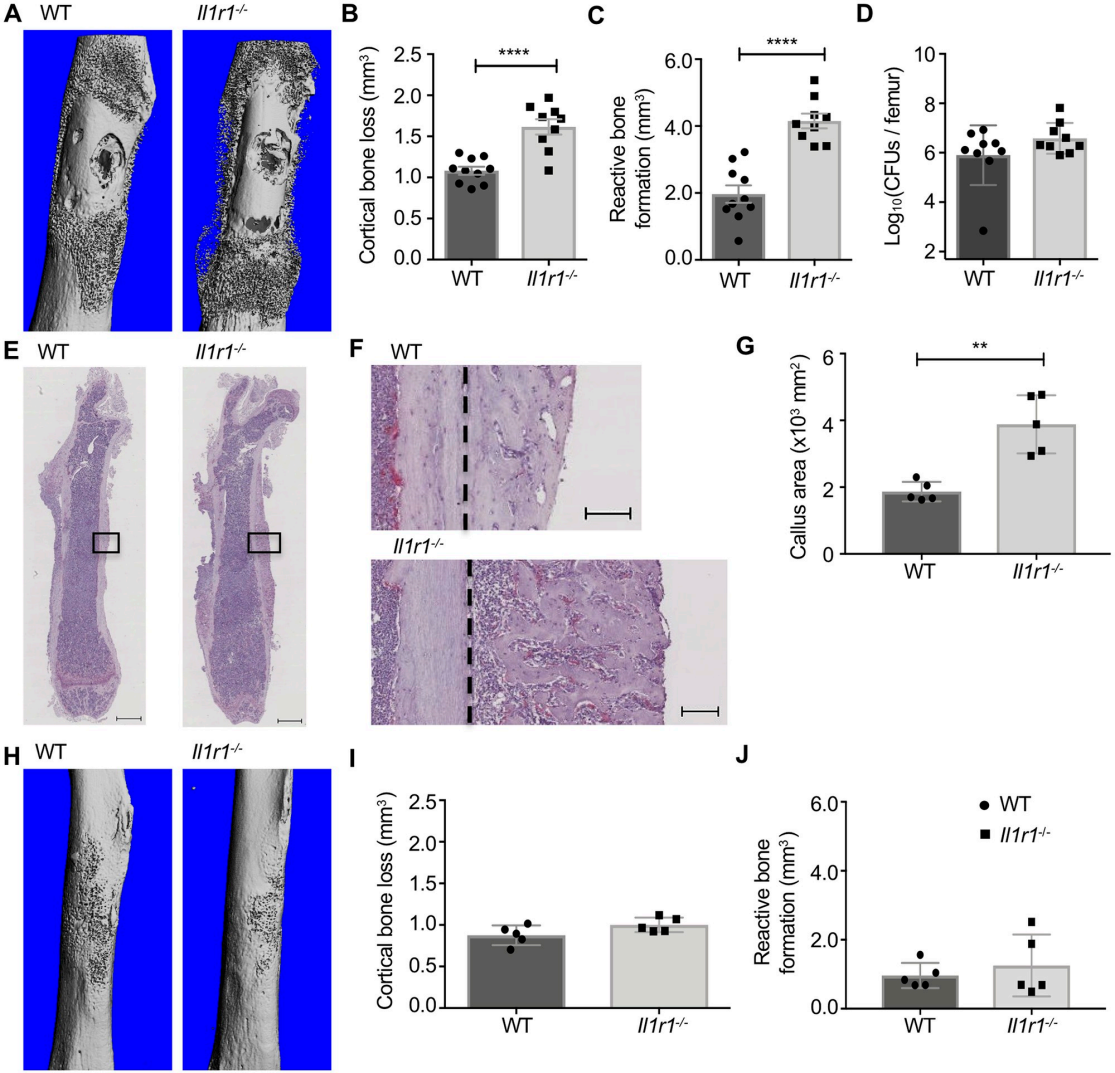
Loss of the IL-1R enhances cortical bone loss and reactive bone formation during *S. aureus* osteomyelitis. (A-J) To determine changes in bone remodeling and final bacterial burdens, female mice were infected with *S. aureus* (10^5^ CFUs: WT *n* = 10, *Il1r1*^-/-^ *n* = 9; duplicate experiments for bone remodeling; WT *n* = 9, *Il1r1*^-/-^ *n* = 9; duplicate experiments for CFU enumeration) or mock infected (WT *n* = 5, *Il1r1*^-/-^ *n* = 5 for bone remodeling) with PBS. Femurs were harvested at day 14 post-infection and scanned using the μCT50. Representative anteroposterior views of *S. aureus* infected femurs (A) and mock infected femurs (H). μCT three-dimensional analysis of cortical bone loss (mm^3^) and reactive bone formation (mm^3^) between *S. aureus* infected (B, C) and mock infected (I, J) WT and *Il1r1*^-/-^ femurs. Bacterial burdens were quantified at day 14 post-infection from WT and *Il1r1*^-/-^ femurs (D). (E, F) After μCT analyses, histologic sections of the *S. aureus* infected femurs were prepared and TRAP-stained. Slides were scanned using a Leica SCN400 Slide Scanner, with representative images shown taken at 0.58X (scale bars = 1 mm) (E) with the region in the black box imaged at 4X (scale bars = 100 μm) (F), with dashed black line demarcating the separation of intact cortical bone (left of dashed line) and the callus (right of dashed line). (G) Tissue IA 2.0 software was used to image callus area of infected femurs at 20X (10^5^ CFUs; n = 5 per genotype). Symbols represent individual data points from each mouse (WT = circles; *Il1r1*^-/-^ = squares), the top line of each bar represents the mean, and error bars represent standard deviation. Unpaired *t*-tests were used to compare CFU burdens, μCT analyses, and Tissue IA 2.0 measurements between WT and *Il1r1*^-/-^ mice. ** *p* < 0.01, **** *p* < 0.0001.

WT mice subjected to *S*. *aureus* osteomyelitis display significant cortical bone destruction and reactive bone formation at the site of infection, while sustaining alterations in osteoclast number and trabecular bone loss in the distal femur (Fig 1A–1I) [6]. Given the important role of IL-1R signaling in skeletal cell differentiation and function *in vitro*, we hypothesized that pathologic bone remodeling during *S*. *aureus* osteomyelitis is mediated, in part, by IL-1R signaling. To test this hypothesis, we first compared changes in cortical bone remodeling between WT and *Il1r1*^*-/-*^ mice using a lower dose (10^5^ CFUs) *S*. *aureus* infection. At the site of infection, *Il1r1*^*-/-*^ mice sustained increased cortical bone loss in comparison to WT mice (Fig 6A and 6B). In areas adjacent to the cortical bone loss, *Il1r1*^*-/-*^ mice had a dramatic increase in new bone formation. In fact, the volume of new bone formation in *Il1r1*^*-/-*^ mice was nearly twice the volume formed in infected WT femurs (Fig 6C). Importantly, these data do not completely control for differences in bacterial burdens at the site of infection, where *Il1r1*^-/-^ mice harbor higher bacterial burdens at day 5 post-infection with a lower inocula (Fig 4E). However, *Il1r1*^-/-^ mice do equilibrate bacterial burdens to the same level at WT mice by day 14 post-infection (Fig 6D). Consistent with an increase in reactive bone formation, histologic analyses revealed increased callus formation in infected *Il1r1*^-/-^ femurs and also demonstrated qualitative differences in callus composition compared to infected WT femurs (Fig 6E–6G). Additionally, the dramatic cortical bone alterations observed in *Il1r1*^*-/-*^ mice were confirmed in littermate controls (S6A and S6B Fig). In contrast to cortical bone remodeling changes during *S*. *aureus* infection, μCT analysis revealed no differences in cortical bone remodeling of a sterile bone defect at day 14 post-surgery, where mock infected WT and *Il1r1*^*-/-*^ femurs had no significant differences in cortical bone loss or reactive bone formation (Fig 6H–6J). Collectively, these data indicate that during *S*. *aureus* osteomyelitis, *Il1r1*^-/-^ mice exhibit significantly altered cortical bone remodeling, with increased reactive bone formation, altered callus architecture, and greater cortical bone loss at the site of infection.

**Fig 7:**
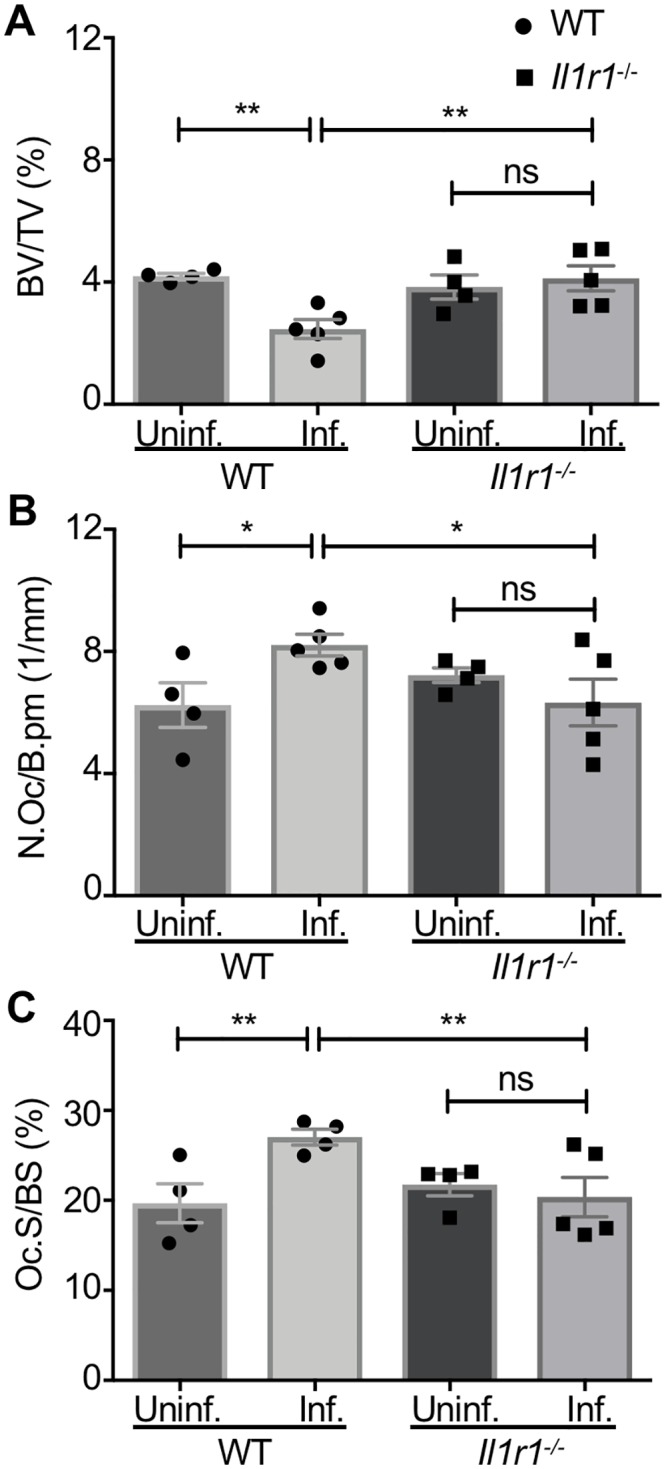
IL-1R contributes to infection-induced osteoclastogenesis during *S. aureus* osteomyelitis. (A-C) Following *S. aureus* infection (10^5^ CFUs), female WT and *Il1r1*^-/-^ infected femurs (*n* = 5 per genotype) and contralateral femurs from infected mice (*n* = 4 per genotype) were harvested, decalcified, processed for sectioning in paraffin, and TRAP-stained for histomorphometric analyses using OsteoMeasure software. Histomorphometric analyses of *S. aureus* infected femurs and contralateral femurs from infected mice were quantified to calculate the trabecular bone volume/total volume (BV/TV) (%) (A), the number of osteoclasts per bone perimeter (N.Oc/B.pm) (1/mm) (B), and the osteoclast surface per bone surface (Oc.S/BS) (%) (C) in the distal femur. Symbols represent individual data points from each mouse (WT = circles; *Il1r1*^-/-^ = squares), the top line of each bar represents the mean, and error bars represent standard deviation. All statistical comparisons used a two-way ANOVA and Fisher’s Least Significant Difference (LSD) test to compare differences in trabecular bone composition of infected and contralateral, uninfected femurs of WT and *Il1r1*^-/-^ mice. * *p* < 0.05, ** *p* < 0.01, ns = not significant.

To elucidate the cellular changes driving differences in bone remodeling between WT and *Il1r1*^-/-^ mice, we next analyzed trabecular bone remodeling during osteomyelitis. Histomorphometric analysis of trabecular bone was performed in both *S*. *aureus* infected femurs and contralateral, uninfected femurs from each genotype. Histomorphometry revealed that the infected femurs from WT mice had significantly lower trabecular BV/TV than contralateral, uninfected femurs (Fig 7A). In contrast, infected femurs from *Il1r1*^-/-^ mice showed no significant differences in BV/TV in comparison to the contralateral, uninfected femur, suggesting that these mice were protected from infection-associated trabecular bone loss despite having significantly higher bacterial burdens in the regions encompassing the trabecular bone over time (Fig 7A; S2 Fig). To determine if differences in osteoclast biology might underlie the distinct trabecular bone remodeling parameters of WT and *Il1r1*^*-/-*^ mice, we calculated the numbers of osteoclasts present on trabecular bone surfaces in both infected and contralateral, uninfected femurs. The infected femurs in WT mice displayed greater osteoclast numbers per bone perimeter (N.Oc/B.pm) and osteoclast surface per bone surface (Oc.S/BS) compared to the contralateral, uninfected femurs, correlating with the infection-induced loss of trabecular bone volume (Fig 7A–7C). In contrast, the infected femurs from *Il1r1*^*-/-*^ mice showed no increase in N.Oc/B.pm or Oc.S/BS when compared to the contralateral, uninfected femur (Fig 7B and 7C). These data suggest that *S*. *aureus* infection causes enhanced osteoclastogenesis in trabecular bone, which is dependent on intact IL-1R signaling and contributes to bone loss.

Histomorphometric analysis revealed that *S*. *aureus* infection enhances osteoclastogenesis and trabecular bone loss in an IL-1R-dependent manner. However, bone volume and remodeling are also significantly impacted by osteoblast function. To determine the contribution of osteoblasts toward altered bone homeostasis and trabecular bone loss during staphylococcal osteomyelitis, we measured bone mineralization in the trabecular bone of infected WT and *Il1r1*^*-/-*^ mice. No differences were observed in mineralizing surface, bone formation rate, or mineral apposition rate between WT and *Il1r1*^*-/-*^ mice (S7A–S7C Fig). These data indicate that IL-1R signaling does not drive differences in trabecular osteoblastic function during *S*. *aureus* infection, and that the decrease in trabecular BV/TV is not a function of decreased osteoblastic bone formation.

### *S*. *aureus* triggers osteoclastogenesis of RANKL-primed myeloid cells through MyD88 and IL-1R signaling

**Fig 8:**
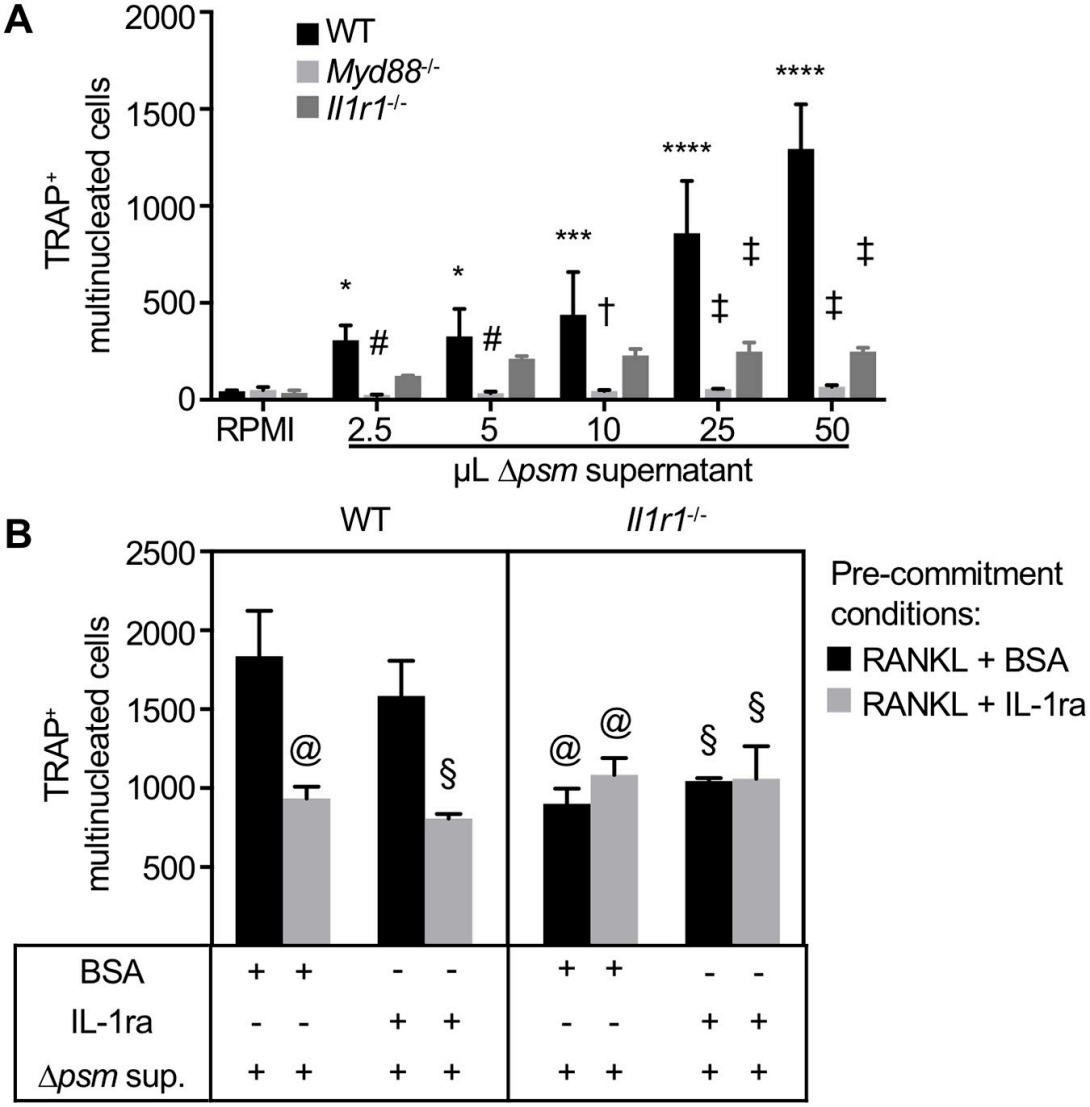
MyD88 and IL-1R signaling drive *S. aureus* enhancement of osteoclast differentiation *in vitro*. (A) Osteoclast precursors were generated from primary WT, *Myd88*^-/-^, and *Il1r1*^-/-^ BMMs by treatment with 35 ng/mL RANKL treatment for 48 hours. After washing 2X with PBS, fresh media was replenished, and cells were stimulated with vehicle (1% casamino acid-supplemented RPMI) or toxin-deficient *S. aureus* supernatant (Δ*psm*) in the absence of ongoing RANKL treatment. All cells were TRAP stained *in vitro* at day 6 and TRAP^+^ multinucleated cells were manually quantified using OsteoMeasure software (*n* = 3 wells per condition, representative of duplicate experiments). (B) WT and *Il1r1*^-/-^ enriched BMMs were stimulated with 35 ng/mL exogenous RANKL for 2 days before stimulating with Δ*psm* supernatants for four days. In order to determine the contribution of IL-1 signaling in this assay, vehicle (0.1% low endotoxin BSA) or recombinant murine IL-1ra (1 μg/mL) were added during the 2 days of RANKL pre-commitment (BSA, black bars; IL-1ra, grey bars) or during the 4 days of Δ*psm* supernatant stimulation (indicated by + under each bar). The top line of each bar represents the mean and error bars represent standard deviation. A repeated measures two-way ANOVA with Dunnett’s multiple comparisons test was performed on cell counts from each genotype, comparing each dose of Δ*psm* supernatant to the vehicle control, where * *p* < 0.05, *** *p* < 0.001, **** *p* < 0.0001, and if not designated by asterisks is non-significant. A repeated measures two-way ANOVA with Tukey’s multiple comparisons test was performed to compare *Myd88*^-/-^ and *Il1r1*^-/-^ TRAP^+^ multinucleated cell counts to WT counts within each Δ*psm* supernatant dose, where # *p* < 0.05, † *p* < 0.001, and ‡ *p* < 0.0001. A three-way ANOVA with Tukey’s multiple comparisons test compared the effects of genotype, IL-1ra treatment during RANKL pre-commitment, and IL-1ra treatment during Δ*psm* supernatant stimulation. Statistical comparisons to WT cells pre-committed with RANKL + BSA and Δ*psm* supernatant + BSA (first black bar from the left) are reported as @ *p* < 0.05, or compared to WT cells pre-committed with RANKL + BSA and Δ*psm* supernatant + IL-1ra (second black bar from the left) are reported as § *p* < 0.05. Comparisons between all RANKL + IL-1ra pre-commitment conditions (grey bars) were non-significant.

Staphylococcal infection causes bone loss and enhanced osteoclastogenesis in trabecular bone. Accordingly, we hypothesized that secreted bacterial factors might augment osteoclast differentiation. To test this hypothesis, we measured osteoclast differentiation of RANKL-primed myeloid progenitors after stimulation with *S*. *aureus* culture supernatant or a vehicle control. To avoid induction of cell death in myeloid cells, we used a *S*. *aureus* strain lacking the alpha-type PSMs, which we previously demonstrated are both necessary and sufficient for causing cell death when staphylococcal supernatants are applied to murine bone marrow-derived macrophages (BMMs) [6, 7]. Stimulation of RANKL-primed BMMs with toxin-deficient supernatant resulted in a dramatic increase in mature osteoclasts, as identified as tartrate resistant acid phosphatase positive (TRAP^+^) multinucleated cells, relative to vehicle control in WT cells (Fig 8A). To determine if this bacterial enhancement of osteoclastogenesis was dependent on IL-1R signaling, we performed similar experiments with *Myd88*^-/-^ and *Il1r1*^-/-^ BMMs. We discovered that *Myd88*^-/-^ and *Il1r1*^-/-^ cells do not undergo robust *S*. *aureus*-mediated osteoclastogenesis, despite being able to differentiate into osteoclasts via canonical RANKL stimulation (Fig 8A, S8A–S8D Fig). Moreover, toxin-deficient *S*. *aureus* supernatants caused significantly less osteoclast formation in *Myd88*^*-/-*^ and *Il1r1*^-/-^ cells in comparison to WT cells (Fig 8A). We next tested the role of IL-1 blockade on osteoclastogenesis using WT or *Il1r1*^*-/-*^ cells, with or without IL-1R antagonist (IL-1ra) treatment. RANKL pre-commitment of WT cells with simultaneous IL-1ra treatment blunted the generation of osteoclast precursors, leading to 50% fewer *S*. *aureus*-stimulated osteoclasts (Fig 8B). This decline in *S*. *aureus*-enhanced osteoclastogenesis results in differentiation to a similar level as is observed in *Il1r1*^-/-^ osteoclast precursors (Fig 8B). These observations indicate that MyD88 and the IL-1R are required for *S*. *aureus*-mediated enhancement of osteoclastogenesis. Therefore, although MyD88 and IL-1R are critical mediators of the anti-staphylococcal immune response, *S*. *aureus* infection also elicits osteoclast-mediated bone loss through MyD88 and IL-1R signaling pathways.

## Discussion

Bacterial osteomyelitis is a debilitating invasive infection of bone that is accompanied by significant damage to skeletal tissues and the surrounding vasculature. Using a model of post-traumatic *S*. *aureus* osteomyelitis, we have detailed dramatic architectural and cellular bone remodeling alterations that accompany *S*. *aureus* infection. In prior research, we determined that some of the cortical bone loss observed during infection is due to *psm-* and *agr*-dependent mechanisms [6]. This is in keeping with the findings of Gillaspy et al., who used a rabbit model of osteomyelitis and observed significantly less bone pathology when infecting with an *agr* mutant [11]. However, the observation of residual bone pathology in mice infected with an *agr* mutant in our osteomyelitis model led us to postulate that host responses to bacterial infection may also contribute to bone loss. The focus of this work was therefore to delineate critical host responses to staphylococci in bone and to elucidate how an innate immune response might impact bone homeostasis.

In this study, we focused primarily on MyD88 and IL-1R signaling cascades given their established roles in innate immune responses against *S*. *aureus* in other models of infection [27–35], in concert with the known effects of these signaling pathways on bone cell function [19–22]. Additionally, patients with single nucleotide polymorphisms (SNPs) in *Myd88*, *Il1a*, and *Il1r1* genes have an increased risk of osteomyelitis and inflammatory joint disorders, further underscoring the importance of these immune pathways in skeletal homeostasis [52–57].

A robust innate immune response was observed in *S*. *aureus* infected femurs, with abundant levels of pro-inflammatory cytokines IL-1α, IL-1β, IL-6, and TNFα detectable as soon as one day after infection, similar to what has been reported in other musculoskeletal infection models [5, 16]. Many cytokines with increased abundance in infected versus mock infected femurs are encoded by IL-1 target genes, including IL-1α, IL-1β, IL-6, MCP-1 (CCL2), and the murine IL-8 homologs, KC (CXCL1) and MIP-2 (CXCL2) [58, 59]. In turn, IL-1 cytokines and IL-6 also promote the release of IL-17 and subsequent G-CSF production, both of which were highly abundant in *S*. *aureus* infected femurs [60]. These data suggest a role for IL-1 signaling in orchestrating downstream inflammatory responses to pathogens in bone. Growth factors and chemokines that support myeloid cell influx and expansion after infection, including M-CSF, G-CSF, MCP-1 (CCL2), MIP-1α (CCL3), MIP-1β (CCL4), KC (CXCL1) and MIP-2 (CXCL2), are also highly abundant in *S*. *aureus* infected femurs relative to mock infected femurs. This observation parallels other reports demonstrating increased levels of myeloid chemokines from osteoblasts after *S*. *aureus* infection, and a role of myeloid chemokines and growth factors in supporting osteoclastogenic bone degradation by promoting expansion of osteoclast precursor cells [61, 62]. Moreover, many of these chemokines have been shown to coordinate neutrophil responses during acute inflammation [63]. These data partially overlap with early cytokine signatures measured in a pin prosthetic implant model of *S*. *aureus* biofilm infection, with IL-1β, IL-6, TNFα, IL-12p70, and IL-17 detected in infected tissues. However local inflammation during biofilm infection was also characterized by increased abundance of IL-2 [64], which was not significantly elevated in our infection model. However, the development of a biofilm has been shown to attenuate the host pro-inflammatory response in a catheter *S*. *aureus* biofilm model, as measured by decreased levels of IL-1β, TNFα, CXCL2, and CCL2 [65]. Future studies should continue to delineate how implant-associated biofilms skew the immune response during osteomyelitis.

The early increase in cardinal pro-inflammatory cytokines after *S*. *aureus* infection indicates that the post-traumatic model of osteomyelitis used in this study is most representative of acute osteomyelitis. However, the bone pathology visualized by day 14 post-infection has clear features suggestive of chronic infection, including significant reactive bone formation and the presence of sequestra [66]. Consistent with a possible shift from acute to chronic infection, we observed production of IFNγ and IL-17 at later time points post-infection, which could represent *S*. *aureus*-specific adaptive Th1/Th17 responses [64]. Delineating the cytokine milieu at later time points after infection will help to more comprehensively characterize the inflammation accompanying osteomyelitis in this model.

Based on the robust early inflammatory responses to *S*. *aureus* in bone coupled with the detection of multiple IL-1 associated cytokines, we focused on the role of MyD88 and IL-1R signaling in coordinating antibacterial defenses during osteomyelitis. Furthermore, since several cardinal pro-inflammatory cytokines, including IL-1, have direct effects on skeletal cell differentiation and function, we hypothesized that MyD88 and IL-1R-dependent signaling pathways would be necessary for control of bacterial proliferation during osteomyelitis, but that these same pathways might also contribute to pathogen-induced bone loss through actions on skeletal cells. To determine the contribution of MyD88 and IL-1R signaling towards antibacterial immune responses during *S*. *aureus* osteomyelitis, we infected mice globally deficient in MyD88, IL-1R, IL-1α, and IL-1β and measured bacterial burdens and morbidity. We found that MyD88 and IL-1R signaling are required to control bacterial burdens in bone in mice infected with a high dose inoculum. Furthermore, even with a lower *S*. *aureus* inoculum, a timecourse experiment revealed that *Il1r1*^-/-^ mice had a delay in ability to control bacterial burdens. In order to determine the relative contributions of IL-1 isoforms to IL-1R-mediated antibacterial immunity in bone, we infected *Il1a*^-/-^ or *Il1b*^-/-^ mice and compared bacterial burdens to those observed in WT and *Il1r1*^-/-^ mice. Mice lacking either IL-1α or IL-1β sustained bacterial burdens not statistically different from bacterial burdens harbored by WT mice, but *Il1a*^-/-^ and *Il1b*^-/-^ mice both harbored significantly less bacterial CFUs than *Il1r1*^-/-^ mice. These data suggest that the loss of both cytokines may be required to recapitulate the enhanced bacterial burdens in *Il1r1*^-/-^ mice. Moreover, the extensive repertoire of innate receptors that signal through MyD88 (e.g. TLRs) likely promote a more effective antibacterial response to *S*. *aureus*, as MyD88 signaling is critical to prevent disseminated disease and death during osteomyelitis.

Our findings that MyD88 and IL-1R mediate antibacterial protection in bone are consistent with data from previous studies demonstrating that *Myd88*^-/-^ and *Il1r1*^-/-^ mice have enhanced susceptibility to bacterial infection in various experimental models [27, 43, 44, 67]. Several studies have also reported that IL-1R signaling contributes to the early influx of neutrophils and abscess formation to protect against *S*. *aureus* cutaneous and prosthetic joint infection [27, 29–31]. Elevated levels of GM-CSF, G-CSF, and CXCL1 in WT mice suggest that neutrophil influx and/or expansion is also a critical early response to *S*. *aureus* in bone to prevent continued bacterial replication and spread. Interestingly, the neutrophilic cytokine response was delayed in *Il1r1*^-/-^ mice in response to *S*. *aureus*, which in congruent with other *Il1r1*^-/-^ mouse models in response to inflammatory stimuli [38–40]. Disorganized abscess architecture in *Il1r1*^-/-^ infected femurs may indicate improper neutrophil mobilization without IL-1R signaling as an underlying mechanism for the inability to control bacterial burdens. Furthermore, we have previously shown that neutrophil depletion leads to significantly increased bacterial burdens during *S*. *aureus* osteomyelitis [7]. Here, we determined that relative neutrophil abundance was lower in *Il1r1*^-/-^ mice at early and late time points in the infected femurs, suggesting that *Il1r1*^*-/-*^ mice have altered systemic neutrophil responses that may correlate with alterations in G-CSF and GM-CSF data. Lower amounts of neutrophils in both the infected and contralateral, uninfected femurs of *Il1r1*^-/-^ mice relative to WT mice support prior observations that *Il1r1*^-/-^ mice have a defect in granulopoiesis [38–40]. Together these reports detail the importance of IL-1R signaling to protect against *S*. *aureus* bone infections by coordinating an effective anti-staphylococcal neutrophil response.

The inability of *Myd88*^-/-^ and *Il1r1*^-/-^ mice to mount appropriate anti-staphylococcal immune responses and the characteristic differences in bone remodeling between WT and *Il1r1*^-/-^ mice led us to confirm these phenotypes with littermate controls bred heterozygously. Contradictory reports have detailed either no difference in bone mass of *Il1r1*^-/-^ mice [68], low bone mass in *Il1r1*^-/-^ mice [69], or greater bone mass in *Il1r1*^-/-^ mice [70, 71] when compared to WT comparators. These studies used various WT comparators (129/J, 129/Sv, BALB/cA, C57BL/6), and also varied with respect to the assessment of mouse age and gender. Additionally, *Myd88*^*-/-*^ mice have altered intestinal barrier function and differences in the microbiome, which can lead to differences in immune function and bone mass [45, 46, 48–51]. Therefore, breeding of heterozygous colonies allowed us to reduce the confounding influence of mouse genotype and microbiome effects to confirm the importance of MyD88 and IL-1R signaling in antibacterial responses and bone remodeling.

To investigate the mechanisms by which *S*. *aureus* alters bone homeostasis to incite bone destruction and reactive bone formation, we measured cortical and trabecular changes in bone architecture via μCT, quantified changes in skeletal cell function and activity *in vivo* using standard bone histomorphometry, and cultured skeletal cells *in vitro* to determine how *S*. *aureus* influences skeletal cell differentiation. These data reveal that *S*. *aureus* osteomyelitis induces changes in bone turnover both locally at the inoculation site, as well as in more distal areas not grossly impacted by abscess formation, leading to the significant loss of cortical and trabecular bone. *Il1r1*^-/-^ mice exhibited more dramatic cortical bone changes, which may be due to differences in bone remodeling processes between WT and *Il1r1*^-/-^ mice, the fact that *Il1r1*^-/-^ mice harbor increased bacterial burdens over the duration of infection, or a combination of these factors. Previous studies have shown that the loss of IL-1R and MyD88 signaling enhances healing of sterile bone defects [72], which may explain the enhanced volume of reactive callus formed on the cortical bone of *Il1r1*^-/-^ mice in our findings. Although we did not observe significant differences in osteoblast-mediated bone parameters in trabecular bone, it is possible that there are significant differences in osteoblast or pre-osteoblast differentiation and function in healing cortical bone (callus).

Trabecular bone is the major site of homeostatic bone remodeling [41], and bone loss here is thought to be multifactorial with potent contributions from inflammation, altered skeletal cell differentiation, and direct interaction with bacterial cells. With respect to the latter mechanisms, we detected viable *S*. *aureus* in the regions of the femur encompassing trabecular bone throughout the course of infection. *S*. *aureus* infection enhanced the number of osteoclasts as well as the actively resorbing trabecular bone surface during osteomyelitis in WT mice, thereby corroborating previous observations of enhanced osteoclast surface from *S*. *aureus* infected human bone biopsies [73]. This may reflect, in part, direct interactions with *S*. *aureus* protein A which induces osteoclastogenesis through TNFR1 and EGFR [10]. However, other bacterial factors that directly enhance osteoclastogenesis *in vivo* remain to be determined. In contrast, *Il1r1*^-/-^ mice were protected from enhanced osteoclastogenesis and trabecular bone loss. Excitingly, although *Il1r1*^-/-^ mice harbored higher bacterial burdens throughout the course of infection, they did not exhibit trabecular bone loss or increased osteoclastogenesis relative to contralateral, uninfected femurs. Taken together, these *in vivo* bone remodeling data indicate that *S*. *aureus* osteomyelitis enhances osteoclastogenesis and triggers trabecular bone loss in WT mice, mainly through IL-1R-dependent effects on osteoclasts.

*In vitro* osteoclast differentiation assays further supported the observation of increased osteoclastogenesis in response to *S*. *aureus in vivo*, as staphylococcal supernatants significantly enhanced osteoclast formation from RANKL-primed WT precursor cells. These data corroborate other reports demonstrating that infection of host cells *in vitro* with live *S*. *aureus* enhances osteoclastogenesis and bone resorbing activity [74], and our *in vivo* data now provide evidence that this enhanced osteoclastogenesis translates to bone loss during infection. Mechanistically, genetic deletion of *Myd88* and *Il1r1* and molecular inhibition of IL-1R signaling were found to confer resistance to *S*. *aureus*-enhanced osteoclastogenesis. Consistent with previously published reports, endogenous IL-1 has been described to promote osteoclastogenesis *in vitro* through synergistic signaling of the IL-1 and RANK receptors in the absence of infection [75]. Canonical osteoclast differentiation is initiated by RANK receptor signaling to activate the transcription factors NFATc1 and cFos, which in turn increase IL-1R expression. This allows IL-1 to signal on osteoclast precursors to potentiate osteoclast formation by activating osteoclast-specific genes, and IL-1 has been reported to enhance “pathologically activated osteoclasts” that favor bone loss [19–22, 70, 75, 76]. In the context of infection, *S*. *aureus* and specific staphylococcal toxins have been found to stimulate the production of IL-1 cytokines [77–79]. Moreover, IL-1 cytokines have been described to promote osteoclastogenesis *in vitro* and lead to bone destruction in murine models of rheumatoid arthritis and autoinflammatory disorders [17, 20, 21, 80, 81]. Therefore, these data are consistent with other observations and suggest that IL-1 signals onto osteoclast precursors to enhance osteoclastogenesis and trabecular bone resorption during infection. However, residual osteoclast formation observed in *Il1r1*^-/-^ cells suggests that while MyD88 is required for osteoclastogenesis in response to staphylococcal supernatant, there are both IL-1R-dependent and independent mechanisms involved. These *in vitro* studies support our findings that a major driver of bone loss during *S*. *aureus* osteomyelitis is coordinated by IL-1R-mediated osteoclast enhancement.

Data presented in this study highlight MyD88 and IL-1R signaling as critical pathways supporting anti-staphylococcal immunity in bone, but also implicate these signaling cascades in promoting bone loss during osteomyelitis. There are a few limitations of the experimental approach outlined in this study. We used globally deficient knockout mice to elucidate how MyD88 and IL-1R signaling impact bone homeostasis and anti-staphylococcal immunity. Given that the adapter protein MyD88 is necessary to relay signals from other upstream receptors including TLRs, future studies should explore the relative contributions of other MyD88-dependent receptors in the pathogenesis of osteomyelitis. In certain *S*. *aureus* infection models, TLR2 and TLR9 have been shown to contribute to anti-staphylococcal immunity [28, 44, 82]. *In vitro* osteoclastogenesis assays imply that other MyD88-dependent receptors can sense and respond to components of *S*. *aureus* in culture to enhance osteoclastogenesis. Accordingly, osteoblast and osteoclast lineage cells are reportedly activated *in vitro* through TLRs and IL-1R signaling to favor bone resorption [18, 83, 84]. During staphylococcal osteomyelitis, it remains unclear how much pathogen-induced bone loss occurs as result of direct osteoclast stimulation versus indirect perturbations of bone homeostasis that involve osteoblasts. This could be tested using MyD88 skeletal cell lineage specific knockout mice. Furthermore, the IL-1R-expressing target cells that stimulate anti-staphylococcal immunity and the source and isoform of IL-1 that promotes bone loss remain unclear.

Collectively, this study details the paradoxical roles of innate immune signaling pathways in the pathogenesis of osteomyelitis. Although MyD88 and IL-1R signaling elicit antibacterial responses during bone infection to protect against bacterial proliferation, dissemination, and systemic disease, they also contribute to host-mediated bone loss. Our findings also highlight a specific MyD88- and IL-1R-dependent mechanism of osteoclast enhancement, thereby uncovering a new mechanism for bone loss during *S*. *aureus* osteomyelitis.

## Materials and methods

### Ethics statement

All experiments involving animals were reviewed and approved by the Institutional Animal Care and Use Committee at Vanderbilt University Medical Center on the animal protocols M12059 and M1800055. All experiments were performed according to NIH guidelines, the Animal Welfare Act, and US Federal law. The murine model of osteomyelitis required inhalational anesthesia with isoflurane (1–5%). Post-operative analgesia (buprenorphine 0.5–0.1 mg/kg) was provided pre-operatively and every 8–12 hours for 48 hours post-infection. Mice were euthanized by CO_2_ asphyxiation with secondary confirmation by cervical dislocation and observation of heart rate and breathing.

### Animal use

C57BL/6J (Stock #: 000664), *Myd88*^*-/-*^ (Stock #: 009088) and *Il1r1*^*-/-*^ (Stock #: 003245) mice were purchased through The Jackson Laboratory. *Il1a*^*-/-*^ and *Il1b*^-/-^ mice were generated as described [85]. WT mice were bred with *Myd88*^*-/-*^ or *Il1r1*^-/-^ mice to produce *Myd88*^*+/-*^ or *Il1r1*^+/-^ mice, respectively. Heterozygous mice were bred together to create mice carrying knockout (-/-), heterozygous (+/-), or wild-type (+/+) alleles for *Myd88* or *Il1r1*. Mice were bred heterozygously to reduce the confounding influence of microbiome effects associated with genotypes and maintenance of separate mouse colonies. The resulting littermates were earpunched and genotyped through Transnetyx, Inc. (Cordova, TN).

### Bacterial strain and growth conditions

All infections were conducted with an erythromycin-sensitive derivative of the USA300 type *S*. *aureus* LAC clinical isolate (AH1263) [86]. The toxin-deficient strain LACΔ*psmα1–4* (Δ*psm*) has been previously described and was used for *in vitro* assays to prevent cell death [6, 87]. Bacteria were routinely grown on tryptic soy agar (TSA) or shaking in tryptic soy broth (TSB) with or without 10 μg/mL erythromycin as detailed previously [6]. To prepare concentrated supernatants, Δ*psm* was grown overnight in RPMI supplemented with 1% casamino acids [7].

### Post-traumatic osteomyelitis infection

The murine model of osteomyelitis was performed as described previously [6, 7]. AH1263 was sub-cultured from an overnight culture, grown for 3 hours, and then adjusted in PBS to a concentration of approximately 1x10^6^ CFUs in 2 μl PBS, unless diluted 1:10 or 1:100 to deliver inoculum doses of 1x10^5^ or 1x10^4^ CFUs, respectively. Osteomyelitis was induced in 5- to 8-week old male and female mice following the introduction of a unicortical bone defect using a 21G needle, into which 2 μl of bacterial suspension or PBS (mock infection) was injected into the intermedullary canal. Muscle fascia and skin were closed with sutures and mice were given buprenorphine analgesic every 12 hours for 48 hours, with daily monitoring until the experimental end point. Mice were euthanized if they met human endpoints, including inability to ambulate, inability to eat or drink, loss of greater than 20% body weight, and/or hunched posture.

### Micro-computed tomography (μCT) of cortical and trabecular bone

Femurs were harvested 14 days post-infection and fixed for 48 hours in neutral buffered formalin at 4°C. Bones were scanned using a μCT50 (Scanco Medical, Switzerland) and analyzed with μCT Tomography V6.3–4 software (Scanco USA, Inc., Wayne, PA). To expand previous μCT50 analyses that assessed only the cortical bone of the femoral diaphysis [6], here the diaphysis and distal epiphysis of each femur were visualized in the scout-view radiographs and imaged with 10.0 μm voxel size at 70 kV, 200 μA, and an integration time of 350 ms in a 10.24 mm view. Each imaging scan resulted in 1088 slices (10.88 mm) of the femur that included the diaphysis surrounding the inoculation site, trabecular bone in the distal femur, and excluded the proximal epiphysis. Three-dimensional volumetric analyses were conducted by contouring transverse image slices in the region of interest. The diaphysis of each femur was comprised of 818 image slices. These image slices were used to quantify cortical bone destruction (mm^3^) and reactive bone formation (mm^3^) surrounding the cortical bone inoculation site as described previously [6]. Trabecular bone measurements were obtained in the distal femur by advancing proximally past the growth plate 30 slices. 101 slices were analyzed with an inclusive contour drawn along the endosteal surface to include trabeculae and exclude the cortical bone. Trabecular bone volume per total volume (%), trabecular number (1/mm), trabecular thickness (mm), and trabecular spacing (mm) were determined by segmentation of the image with a lower threshold of 329 mg HA/ccm, sigma 1.3, and support 1.

### Bone histology and histomorphometric analysis of osteoclasts in trabecular bone

After μCT imaging, femurs were decalcified for three days in 20% EDTA at 4°C. Decalcified bones were processed and embedded in paraffin before sectioning at 4μm thickness through the infectious nidus and bone marrow cavity using a Leica RM2255 microtome. Sectioned femurs were stained with a modified hematoxylin and eosin (H&E) that included orange G and phloxine for enhanced bone contrast, tartrate-resistant acid phosphatase (TRAP) stain with hematoxylin counterstain, or 3,3’-diaminobenzidine (DAB) immunohistochemistry to detect myeloperoxidase (MPO). OsteoMeasure software (OsteoMetrics, Inc., Decatur, GA) was used to manually analyze TRAP-stained histologic sections at a region of interest encompassing the trabeculae proximal to the growth plate in the distal femur. Osteoclast number, osteoclast surface, and bone perimeter were calculated and reported per ASBMR standards [42]. A Leica SCN400 Slide Scanner was used to scan stained femur sections in brightfield at 20X. Images were uploaded to and imaged with the Digital Imaging Hub (Leica Biosystems, Buffalo Grove, IL) and Tissue Image Analysis 2.0 (Tissue IA 2.0) (Leica Microsystems, Buffalo Grove, IL) was used to analyze callus area of infected femurs at 20X.

### Determination of bone formation rate with double calcein labelling

WT and *Il1r1*^-/-^ mice were intraperitoneally injected with 20 mg/kg calcein on days 8 and 12 post-infection with 10^5^ CFUs. Femurs were subsequently harvested, formalin fixed, and dehydrated prior to embedding in poly(methyl methacrylate) for sectioning, and counterstained with toluidine blue. Fluorescent labels were identified as single- or double-labeled surface. Fluorescent labels and trabecular bone were traced in the distal femur using OsteoMeasure software, and the mineralizing surface per bone surface (MS/BS), mineral apposition rate (MAR), and bone formation rate per bone surface (BFR/BS) were calculated per ASBMR standards [42].

### CFU enumeration

At various time points post-infection, tissues were harvested and homogenized using a BulletBlender and NAVY lysis tubes (Next Advance, Inc., Averill Park, NY) at 4°C. To enumerate bacterial CFUs in infected femurs, the whole femur or only the regions encompassing the trabecular bone (i.e. metaphyses and epiphyses) were homogenized in PBS. To maximize cytokine signals in femur homogenates, CelLytic Buffer MT Cell Lysis Reagent (Sigma, Saint Louis, MO) was substituted for PBS to specifically lyse mammalian cells. Livers and kidneys were homogenized in PBS. Femur and organ homogenates were vortexed, serially diluted in PBS, and plated on TSA for bacterial enumeration. Femur homogenates lysed in CelLytic Buffer MT and PBS showed no difference in recoverable bacterial burdens.

### Multiplexed cytokine detection

Following homogenization, femur homogenates were centrifuged at 4000 x g for 5 minutes to remove debris and the supernatant was stored at -80°C for subsequent analysis using Milliplex MAP multiplex magnetic bead-based antibody detection kits (EMD Millipore, Billerica, MA) according to the manufacturer’s protocols. Cytokine quantification from bone homogenates was performed using the 32-plex Mouse Cytokine/Chemokine Magnetic Bead Panel (MCYTMAG-70K-PX32) on the FLEXMAP 3D instrument. The quality controls for IL-13 failed, and these data were excluded.

Cytokine levels from femurs homogenized in 500 μl volume were read as pg/mL. Femur homogenates reported as relative values were homogenized in PBS, whereas cytokines values corrected for total protein were homogenized in CelLytic Buffer MT to maximize cytokine signals. Total protein (mg/mL) was quantified using the Pierce BCA Protein Assay Kit (ThermoFisher Scientific, Waltham, MA) per manufacturer’s directions. Infected *Il1r1*^-/-^ femurs were up to two times larger than WT infected femurs and four times larger than mock infected WT and *Il1r1*^-/-^ femurs. Cytokine levels are therefore reported as pg cytokine/mg protein to control for femur size differences between infected *Il1r1*^-/-^ and WT femurs.

### Flow cytometry

Following *S*. *aureus* infection (10^5^ CFUs), femurs from WT and *Il1r1*^-/-^ mice were harvested at 1, 3, 5, and 14 days post-infection. Whole bone marrow (WBM) was flushed through a 70 μm nylon cell strainer (Falcon, Corning, New York) and red blood cells (RBCs) were lysed using the Ammonium Chloride Potassium (ACK) Lysing Buffer (Lonza, Walkersville, MD). Bone marrow (BM) mononuclear cells were counted and 1 million cells were plated per well and washed in PBS supplemented with 3% FBS and 0.1% sodium azide (FACS buffer). Cells were incubated with Anti-CD16/CD32 (Biolegend, 1:100, clone 93, San Diego, CA) to block non-specific antibody staining. Cells were then incubated with a mixture of murine-specific cell surface antibodies on ice, including Anti-Ly6G-PE (Biolegend, 1:3200, clone 1A8), Anti-Ly6C-PE-Dazzle 594 (Biolegend, 1:1600, clone HK1.4), Anti-CD68-PE-Cy7 (Biolegend, 1:100, clone FA-11), Anti-CD11b-APC (Tonbo 1:4800, clone M1/70, San Diego, CA), and Anti-CD45-AlexaFluor 700 (Biolegend, 1:400, clone 30-F11). Cells were washed two times in FACS buffer, resuspended in 2% paraformaldehyde solution, and run on a 3-laser BD LSRII flow cytometer the following day. Single BM cells were identified from successive gates, including side scatter-area by forward scatter-area (SSC-A x FSC-A), forward scatter area by height (FSC-A x FSC-H), and side scatter area by height (SSC-A x SSC-H). Next, CD45^+^ cells, CD11b^+^ cells, and Ly6G^+^LyC^lo^ cells (neutrophils) were gated sequentially.

### Osteoclastogenesis assays

WBM was flushed from femurs of 8- to 13-week old male mice using α-MEM media. Following RBC lysis, WBM was resuspended in a 90% FBS and 10% DMSO solution and frozen in liquid nitrogen until thawed for use. BMMs were enriched by plating 8 to 13 million cells per 10 cm dish in α-MEM, 10% FBS, 1X Penicillin/Streptomycin (P/S), and 100 ng/mL recombinant murine M-CSF (R&D Systems, Minneapolis, MN, 416-ML) for 4 days. Non-adherent cells were removed, and adherent cells were washed with PBS, scraped into fresh media, and counted prior to plating. Enriched BMMs were plated at a density of 50,000 cells/well in 96-well plates, and media (α-MEM, 10% FBS, 1X P/S) was supplemented 1:20 with CMG14-12 as an M-CSF source [88]. Osteoclastogenesis assays were performed with RANKL-primed osteoclast precursors, which were generated by plating BMMs in 35 ng/mL recombinant murine RANKL (R&D Systems, Minneapolis, MN, 462-TR) for 2 days. Prior to stimulation, RANKL-primed osteoclast precursors were washed twice with PBS. RANKL-primed osteoclast precursors were stimulated with either a vehicle control (1% casamino acid-supplemented RPMI) or Δ*psm* supernatant. M-CSF was supplemented into the media containing each stimulation. To test the specific role of IL-1R inhibition on *S*. *aureus*-enhanced osteoclast differentiation, osteoclastogenesis assays in WT and *Il1r1*^-/-^ cells were conducted with the addition of a vehicle control (0.1% low endotoxin BSA) or 1 μg/mL recombinant murine IL-1ra (Novus Biologicals, LLC, Littleton, CO, NBP2-35105) during the 2 days of RANKL pre-commitment or during the 4 days of Δ*psm* supernatant stimulation. On day 6 in culture, all RPMI- and *S*. *aureus*-stimulated osteoclastogenesis assays were fixed with a 4% formaldehyde and 0.05% Triton X-100 solution in PBS (10 minutes) and 1:1 acetone:ethanol (1 minute), before TRAP staining with reagents from the Acid Phosphatase, Leukocyte (TRAP) Kit (Sigma, Saint Louis, MO, 378A). In control osteoclastogenesis assays without *S*. *aureus* supernatant stimulation, cells were stimulated at the time of plating with 1:20 CMG14-12 and 35ng/mL RANKL. Fresh media, CMG14-12, and RANKL were replenished on days 4 and 6 in culture, with cells TRAP stained on day 7. OsteoMeasure was used to manually quantify mature osteoclasts, identified as TRAP^+^ multinucleated cells.

### Statistical analysis

Data analysis and statistical tests were conducted using Graph Pad Prism software. Unpaired *t*-tests were used to compare CFU burdens, measurements of bone architecture using μCT and histology, cytokine levels, neutrophil abundance, and TRAP^+^ multinucleated cell counts when two groups were being compared. Log-rank Mantel Cox tests compared survival curves between genotypes for each *S*. *aureus* inoculum. A one-way ANOVA with Tukey’s multiple comparison test compared CFU burdens from femurs between multiple genotypes. A two-way ANOVA was used with Fisher’s Least Significant Difference (LSD) test to compare the effects of genotype and infection status between histomorphometry measurements. A repeated measures two-way ANOVA with Tukey’s multiple comparisons test was used to compare TRAP^+^ multinucleated cell counts between genotype at each Δ*psm* supernatant dose. Repeated measures two-way ANOVAs with Dunnett’s multiple comparisons test were conducted on TRAP^+^ cell counts from each genotype, to compare Δ*psm* supernatant dosage effects. A three-way ANOVA with Tukey’s multiple comparisons test was used to compare cell genotype, IL-1ra pre-treatment, and IL-1ra treatment alongside Δ*psm* supernatant stimulation. *P* values of less than 0.05 were considered statistically significant. Details on number of data points, experimental replicates, calculated standard deviation, and statistical significance for each experiment are described in figure legends.

## Supporting information

S1 Fig Cortical and trabecular bone architecture of *S*. *aureus* infected femurs via histology. **(A-C)** Femurs were harvested from female WT mice (*n* = 5) 14 days after *S*. *aureus* infection (10^6^ CFUs). Representative modified H&E section of an infected female WT femur, imaged at 0.58X (scale bar = 1mm) **(A)** shown with a grey box surrounding the central portion of the diaphysis and the extent of abscess formation, and a black box surrounding trabecular bone in the distal femur as imaged at 1.28X (scale bar = 1 mm) **(B, C)**. **(B)** Diaphysis and medullary cavity as outlined in the grey box, showing abscesses as indicated by white arrows and a *S*. *aureus* microcolony by a grey arrow. **(C)** Distal femur containing trabecular bone as outlined in the black box.
(TIF)

S2 Fig *S*. *aureus* burdens from femoral metaphyses and epiphyses during *S*. osteomyelitis. Femurs were harvested from female WT and *Il1r1*^-/-^ mice at days 1, 3, 5, and 14 days after *S*. *aureus* infection (10^5^ CFUs) (*n* = 3 per genotype). Distal and proximal femoral epiphyses were homogenized to quantify bacterial burdens in areas encompassing trabecular bone. *S*. *aureus* CFUs were detectable in the ends of WT and *Il1r1*^-/-^ femurs at all time points. Symbols represent individual data points from each mouse (WT = circles; *Il1r1*^*-/-*^ = squares), the top of each bar represents the mean, and error bars represent the standard deviation. Multiple *t*-tests were used to compare CFU burdens between WT and *Il1r1*^-/-^ mice at each time point. ** *p* < 0.01.
(TIF)

S3 Fig *Myd88*^-/-^ mice euthanized at humane endpoints exhibited greater *S*. *aureus* dissemination compared to WT mice. Following infection with 10^5^ *S*. *aureus* CFUs, female *Myd88*^*-/-*^ mice that lost greater than 20% of their body weight were humanely euthanized at day 8 and day 9 post-infection with a randomly chosen female WT comparator (*n* = 2 per genotype) to compare bacterial burdens enumerated from the infected femurs, and to determine dissemination to the kidneys and liver. Symbols represent individual data points from each mouse (WT = circles; *Myd88*^*-/-*^ = squares), the top of each bar represents the mean, and error bars represent the standard deviation. Unpaired *t*-tests were used to compare CFU burdens between WT and *Myd88*^*-/-*^ organ homogenates. ** *p* < 0.01, ns = not significant.
(TIF)

S4 Fig MyD88 protects against local *S*. *aureus* burdens and dissemination during *S*. *aureus* osteomyelitis in male and female *Myd88*^*+/+*^ and *Myd88*^*-/-*^ littermates. **(A-C)** *Myd88*^*+/-*^ mice were bred to produce *Myd88*^*+/+*^ and *Myd88*^*-/-*^ littermate controls. Male (*n* = 5 each genotype) and female (*n* = 5 *Myd88*^*+/+*^; *n* = 3 *Myd88*^*-/-*^) littermate controls were infected with 10^6^ *S*. *aureus* CFUs to establish osteomyelitis. All groups were monitored for severe weight loss and signs of sepsis. **(A)** Male *Myd88*^*+/+*^ (*n* = 5, black) and *Myd88*^*-/-*^ (*n* = 2, blue) mice survived until day 14. Log-rank Mantel Cox test was used to compare male *Myd88*^*+/+*^ and *Myd88*^*-/-*^ survival curves due to infection mortality. * *p* < 0.05. **(B, C)** Bacterial burdens were enumerated from the infected femur, kidneys, and liver from male **(B)** and female **(C)** mice at day 14 post-infection. Symbols represent individual data points from each mouse (*Myd88*^*+/+*^ = circles; *Myd88*^*-/-*^ = squares), the top line of each bar represents the mean and error bars represent standard deviation. Unpaired *t*-tests were used to compare CFU burdens between *Myd88*^*+/+*^ and *Myd88*^*-/-*^ organ homogenates. * *p* < 0.05, ** *p* < 0.01, ns = not significant.
(TIF)

S5 Fig Flow cytometry gating scheme for identification of neutrophils. **(A-F)** WT and *Il1r1*^-/-^ mice were infected with 10^5^ *S*. *aureus* CFUs and at days 1, 3, 5, and 14 days after infection, the infected and contralateral, uninfected femurs were harvested, and BM was collected for flow cytometry. Data shown here represent the gating scheme for each sample at each time point, where labels on plots can be identified by SSC = side scatter, FSC = forward scatter, A = area, H = height, or cellular marker conjugated to a fluorophore. BM cells were identified **(A)**, followed by two single-cell gates **(B, C)**, identification of CD45^+^ cells **(D)**, CD11b^+^ cells **(E)**, and finally the neutrophil population represented in quadrant 3 (Q3) as Ly6G^+^Ly6C^lo^ **(F)**.
(TIF)

S6 Fig *Il1r1*^*-/-*^ mice exhibit enhanced cortical bone loss and reactive bone formation relative to *Il1r1*^+/+^ littermates during *S*. *aureus* osteomyelitis. **(A, B)** *Il1r1*^+/+^ and *Il1r1*^*-/-*^ littermate female mice were infected with 10^6^ *S*. *aureus* CFUs (*Il1r1*^+/+^ *n* = 5, *Il1r1*^*-/-*^ *n* = 4) to assess changes in cortical bone architecture. Femurs were harvested at day 14 post-infection and were scanned using the μCT50. **(A, B)** Cortical bone loss (mm^3^) **(A)** and reactive bone formation (mm^3^) **(B)** from infected *Il1r1*^*+/+*^ *and Il1r1*^-/-^ femurs were quantified using μCT analysis. Symbols represent individual data points from each mouse (*Il1r1*^+/+^ = circles, *Il1r1*^*-/-*^ = squares), the top line of each bar represents the mean, and error bars represent standard deviation. Unpaired *t*-tests were used to compare cortical bone loss and reactive bone formation between *Il1r1*^+/+^ and *Il1r1*^*-/-*^ mice. * *p* < 0.05, ** *p* < 0.01.
(TIF)

S7 Fig WT and *Il1r1*^-/-^ mice have similar bone formation rates and osteoblast activity in trabecular bone during *S*. *aureus* osteomyelitis. **(A-C)** Female mice were infected with 10^5^ *S*. *aureus* CFUs (WT *n* = 5, *Il1r1*^*-/-*^ *n* = 4), with 20 mg/kg calcein injected intraperitoneally on days 8 and 12 post-infection. Femurs were harvested at day 14 post-infection, and embedded in poly(methyl methacrylate) for sectioning. Calcein incorporated into the trabecular bone, and single- and double-labeled fluorescent surfaces were traced relative to total bone surface using OsteoMeasure software. **(A-C)** OsteoMeasure software was used to calculate mineralizing surface per bone surface (MS/BS) (%) **(A)**, bone formation rate per bone surface (BFR/BS) **(B)**, and mineral apposition rate (MAR) **(C)** from WT and *Il1r1*^*-/-*^ infected femurs. Symbols represent individual data points from each mouse (WT = circles, *Il1r1*^*-/-*^ = squares), the top line of each bar represents the mean, and error bars represent standard deviation. Unpaired *t*-tests were used to compare measurements of osteoblast activity *in vivo* between infected WT and *Il1r1*^*-/-*^ mice. ns = not significant.
(TIF)

S8 Fig WT, *Myd88*^-/-^, and *Il1r1*^-/-^ cells undergo RANKL-mediated osteoclastogenesis at similar levels. **(A-D)** WT, *Myd88*^*-/-*^, and *Il1r1*^*-/-*^ BMMs were plated at 50,000 cells per well in a 96-well plate. Cell cultures were supplemented with 35 ng/mL RANKL and 1:20 CMG14-12 supernatant as an M-CSF source. Media and reagents were replenished on days 4 and 6 in culture (i.e. RANKL stimulation was continued for the entire experiment), and cells were fixed and stained for TRAP expression on day 7. **(A-D)** Cells were imaged at 10X **(A, C)** and TRAP^+^ multinucleated cells were counted using the OsteoMeasure software **(B, D)**. Symbols represent individual well counts from (WT = circles, *Myd88*^*-/-*^ and *Il1r1*^*-/-*^ = squares), the top of each bar represents the mean, and error bars represent standard deviation. Unpaired *t*-tests were used to compare cell counts between WT and *Myd88*^*-/-*^ or *Il1r1*^*-/-*^ cells. ns = not significant.
(TIF)

S1 Table Cytokine levels in WT and *Il1r1*^-/-^ mice during *S*. *aureus* osteomyelitis. Femurs from female WT and *Il1r1*^-/-^ mice were harvested at days 1, 3, 5, 10, and 14 post-infection with 10^5^ *S*. *aureus* CFUs (*n* = 3 mice per timepoint). Femurs were homogenized in CelLytic Buffer MT and the supernatant from the bone homogenate was analyzed using a 32-plex Millipore kit on the Luminex platform to obtain cytokine abundance. The protein content in the femur lysate was quantified using a BCA kit. Cytokine data are reported as the mean pg cytokine/mg protein ± standard deviation, and were compared between infected WT and *Il1r1*^-/-^ mice using multiple *t*-tests. * *p* < 0.05, ** *p* < 0.01, *** *p* < 0.001.
(DOCX)
