# Supplementary figures and images for: MyD88 and IL-1R signaling drive antibacterial immunity and osteoclast-driven bone loss during Staphylococcus aureus osteomyelitis
Source: PLoS Pathog. 2019 Apr 12;15(4):e1007744. doi: 10.1371/journal.ppat.1007744 (PMC6481883; doi:10.1371/journal.ppat.1007744)

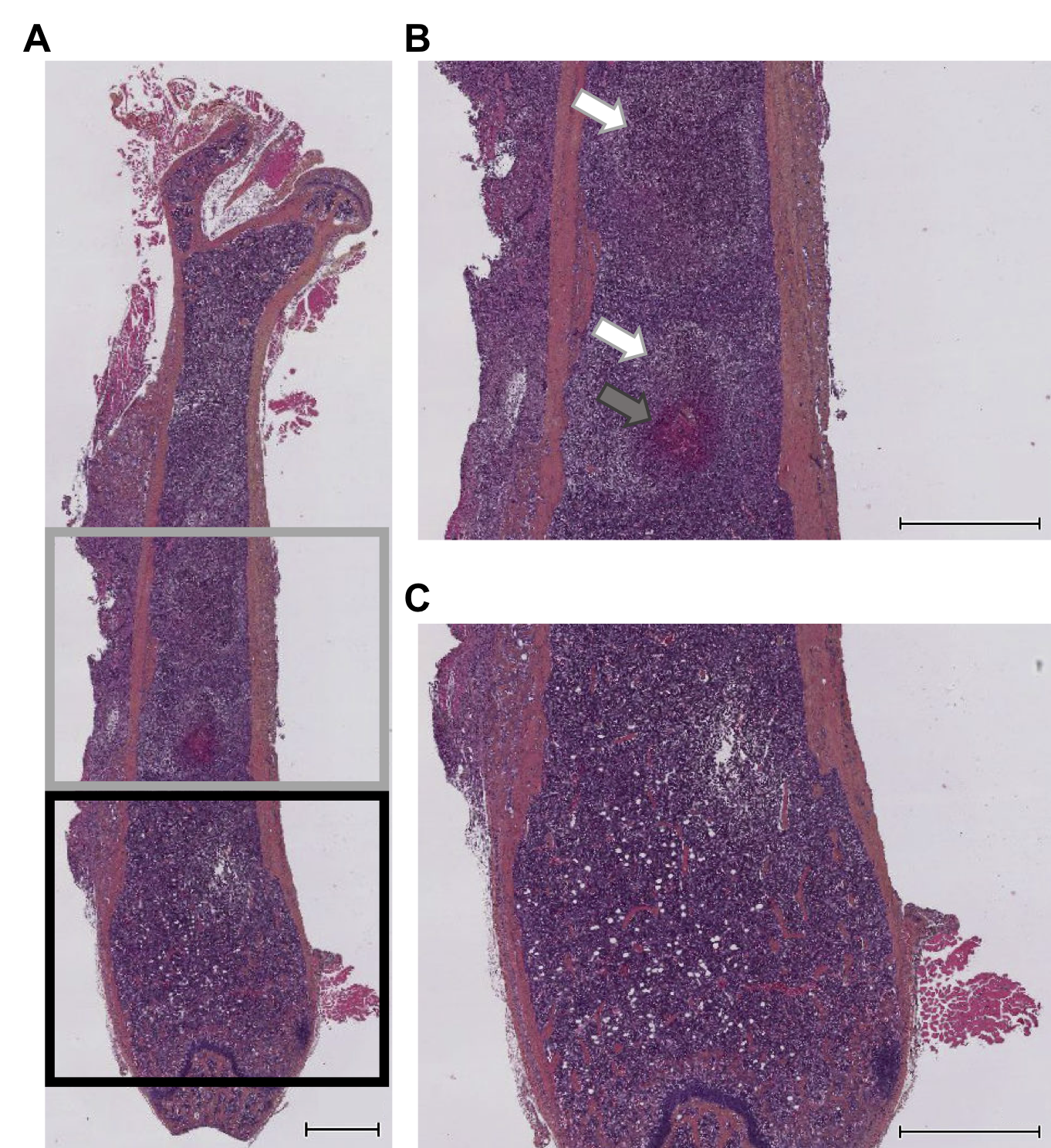

Supplement: S1 Fig — (A-C) Femurs were harvested from female WT mice (n = 5) 14 days after S. aureus infection (106 CFUs). Representative modified H&E section of an infected female WT femur, imaged at 0.58X (scale bar = 1mm) (A) shown with a grey box surrounding the central portion of the diaphysis and the extent of abscess formation, and a black box surrounding trabecular bone in the distal femur as imaged at 1.28X (scale bar = 1 mm) (B, C). (B) Diaphysis and medullary cavity as outlined in the grey box, showing abscesses as indicated by white arrows and a S. aureus microcolony by a grey arrow. (C) Distal femur containing trabecular bone as outlined in the black box. (TIF) [file ppat.1007744.s001.tif]

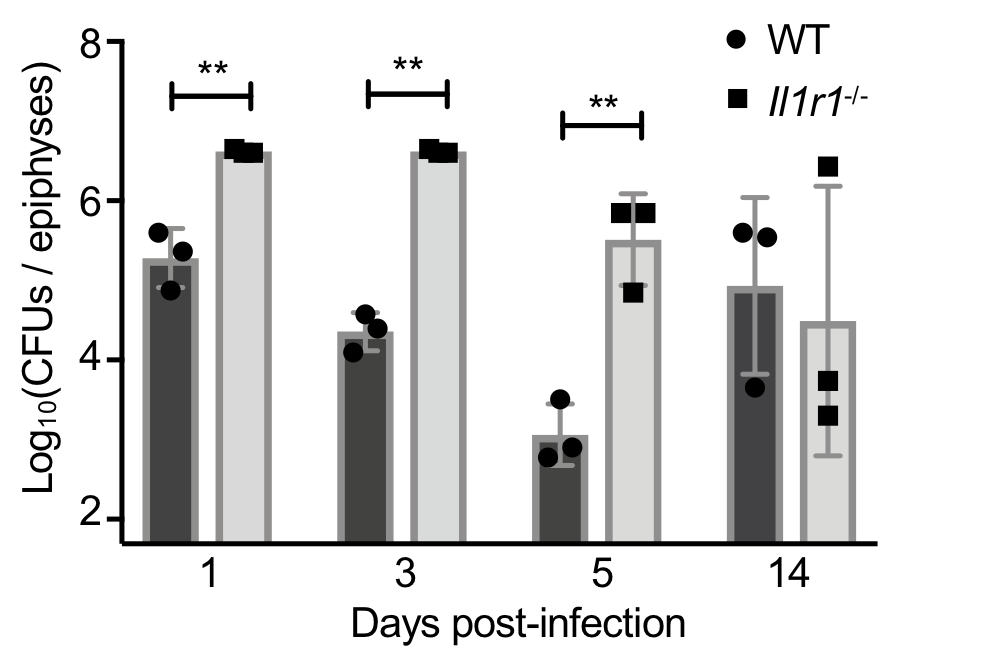

Supplement: S2 Fig — Femurs were harvested from female WT and Il1r1-/- mice at days 1, 3, 5, and 14 days after S. aureus infection (105 CFUs) (n = 3 per genotype). Distal and proximal femoral epiphyses were homogenized to quantify bacterial burdens in areas encompassing trabecular bone. S. aureus CFUs were detectable in the ends of WT and Il1r1-/- femurs at all time points. Symbols represent individual data points from each mouse (WT = circles; Il1r1-/- = squares), the top of each bar represents the mean, and error bars represent the standard deviation. Multiple t-tests were used to compare CFU burdens between WT and Il1r1-/- mice at each time point. ** p < 0.01. (TIF) [file ppat.1007744.s002.tif]

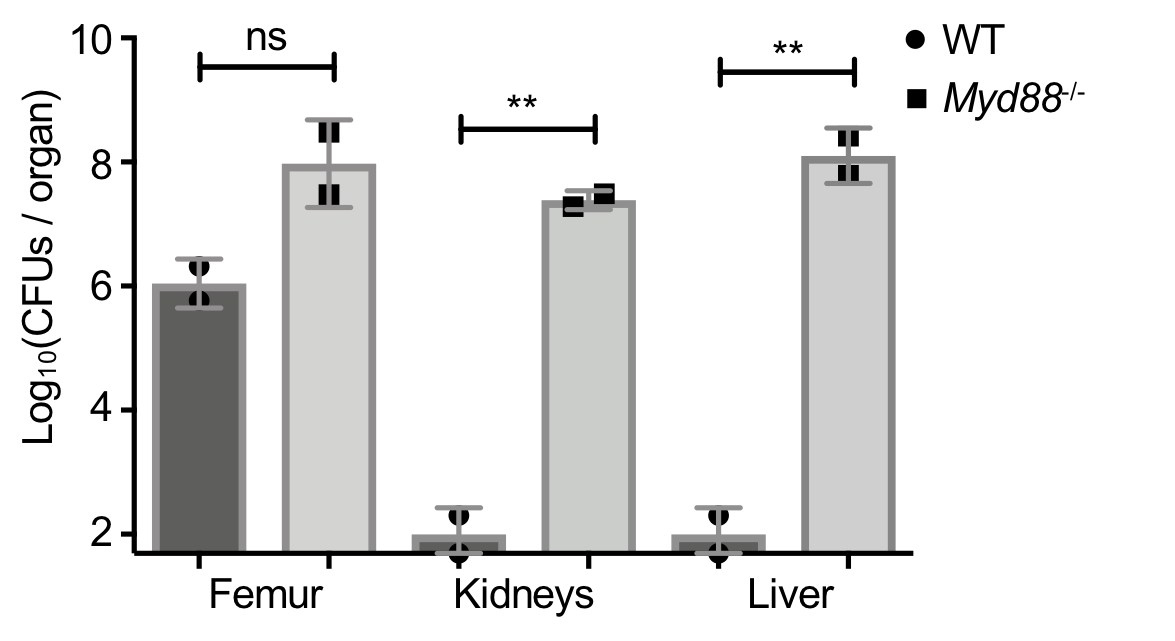

Supplement: S3 Fig — Following infection with 105 S. aureus CFUs, female Myd88-/- mice that lost greater than 20% of their body weight were humanely euthanized at day 8 and day 9 post-infection with a randomly chosen female WT comparator (n = 2 per genotype) to compare bacterial burdens enumerated from the infected femurs, and to determine dissemination to the kidneys and liver. Symbols represent individual data points from each mouse (WT = circles; Myd88-/- = squares), the top of each bar represents the mean, and error bars represent the standard deviation. Unpaired t-tests were used to compare CFU burdens between WT and Myd88-/- organ homogenates. ** p < 0.01, ns = not significant. (TIF) [file ppat.1007744.s003.tif]

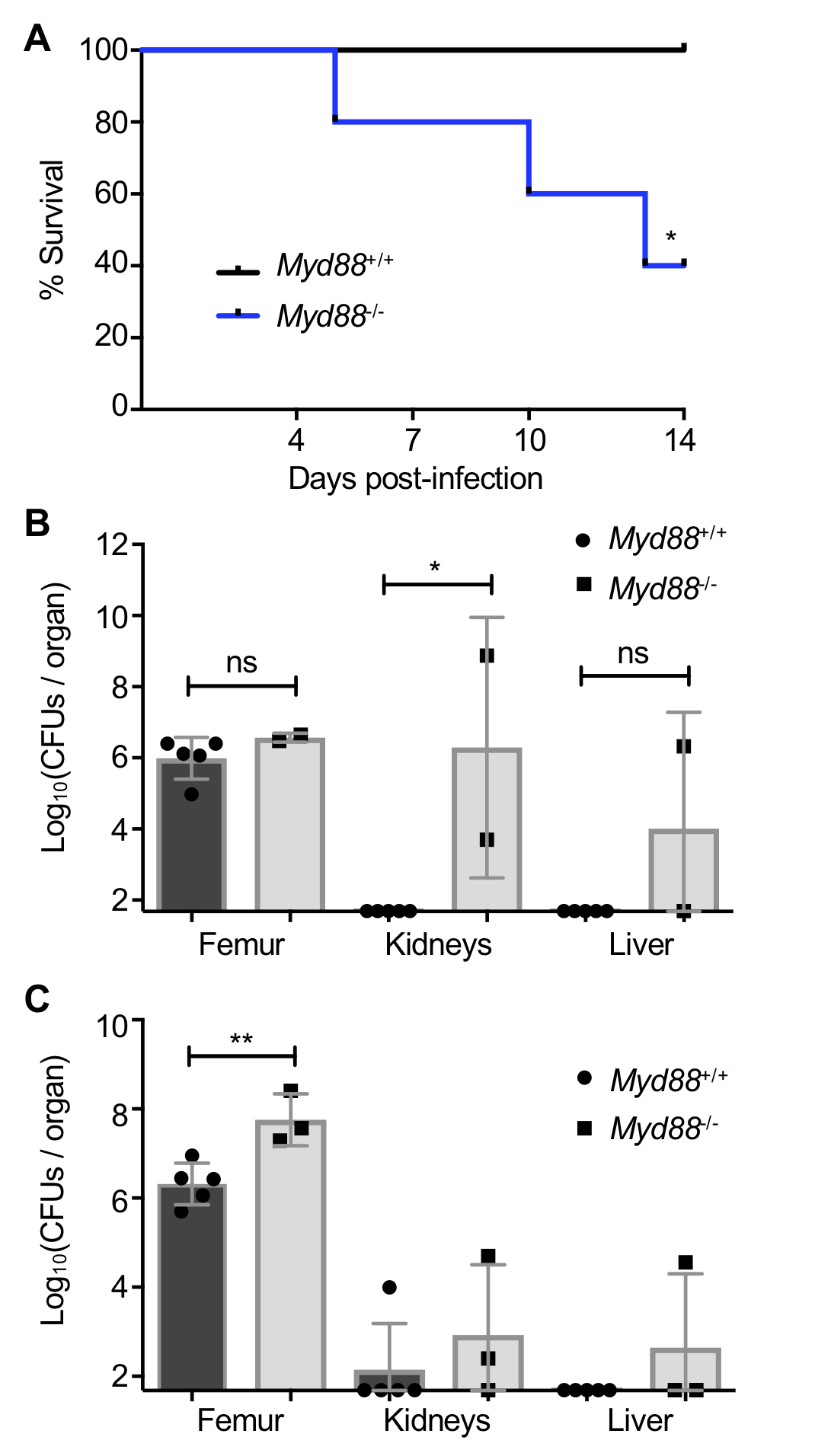

Supplement: S4 Fig — (A-C) Myd88+/- mice were bred to produce Myd88+/+ and Myd88-/- littermate controls. Male (n = 5 each genotype) and female (n = 5 Myd88+/+; n = 3 Myd88-/-) littermate controls were infected with 106 S. aureus CFUs to establish osteomyelitis. All groups were monitored for severe weight loss and signs of sepsis. (A) Male Myd88+/+ (n = 5, black) and Myd88-/- (n = 2, blue) mice survived until day 14. Log-rank Mantel Cox test was used to compare male Myd88+/+ and Myd88-/- survival curves due to infection mortality. * p < 0.05. (B, C) Bacterial burdens were enumerated from the infected femur, kidneys, and liver from male (B) and female (C) mice at day 14 post-infection. Symbols represent individual data points from each mouse (Myd88+/+ = circles; Myd88-/- = squares), the top line of each bar represents the mean and error bars represent standard deviation. Unpaired t-tests were used to compare CFU burdens between Myd88+/+ and Myd88-/- organ homogenates. * p < 0.05, ** p < 0.01, ns = not significant. (TIF) [file ppat.1007744.s004.tif]

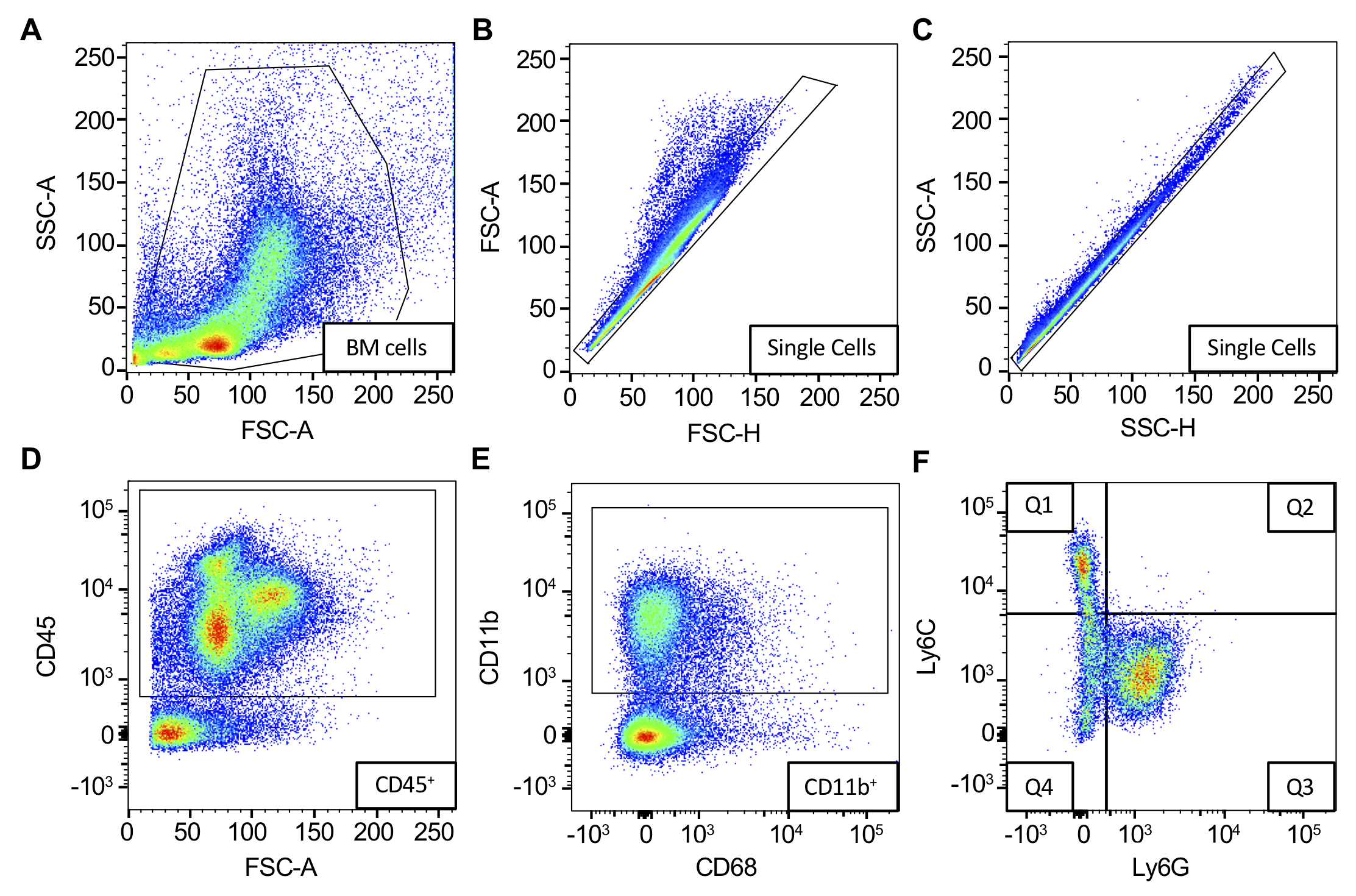

Supplement: S5 Fig — (A-F) WT and Il1r1-/- mice were infected with 105 S. aureus CFUs and at days 1, 3, 5, and 14 days after infection, the infected and contralateral, uninfected femurs were harvested, and BM was collected for flow cytometry. Data shown here represent the gating scheme for each sample at each time point, where labels on plots can be identified by SSC = side scatter, FSC = forward scatter, A = area, H = height, or cellular marker conjugated to a fluorophore. BM cells were identified (A), followed by two single-cell gates (B, C), identification of CD45+ cells (D), CD11b+ cells (E), and finally the neutrophil population represented in quadrant 3 (Q3) as Ly6G+Ly6Clo (F). (TIF) [file ppat.1007744.s005.tif]

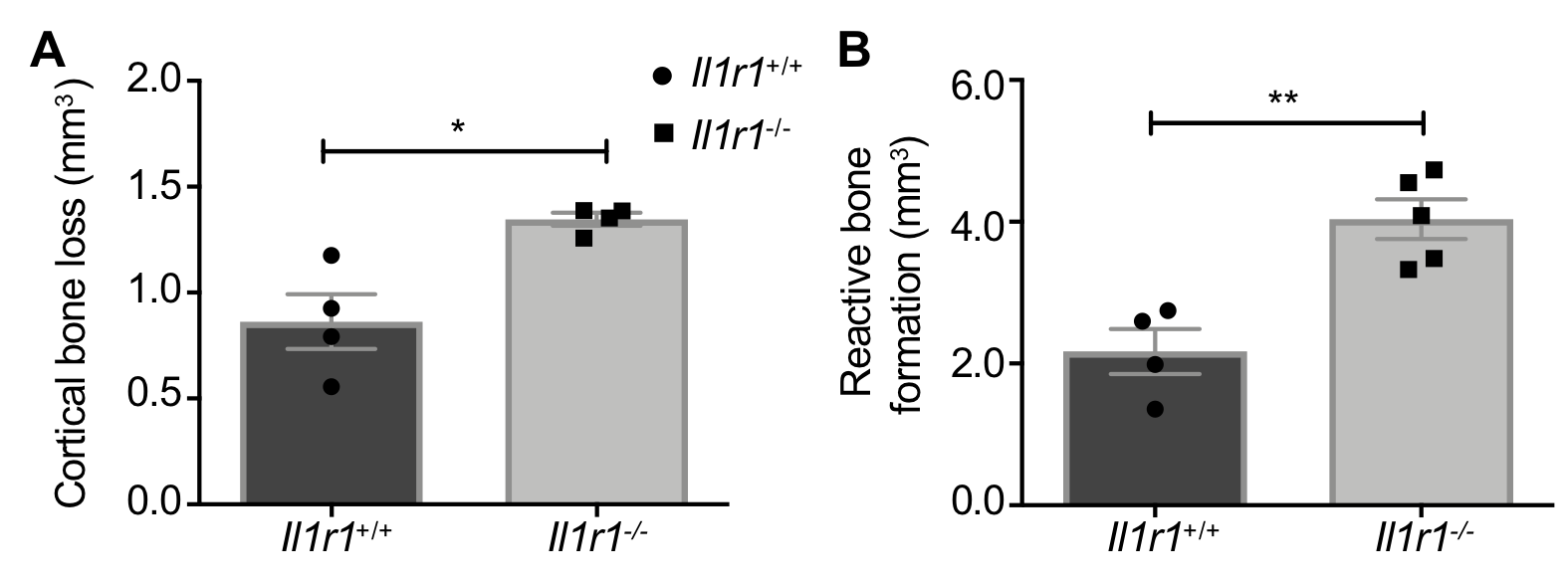

Supplement: S6 Fig — (A, B) Il1r1+/+ and Il1r1-/- littermate female mice were infected with 106 S. aureus CFUs (Il1r1+/+ n = 5, Il1r1-/- n = 4) to assess changes in cortical bone architecture. Femurs were harvested at day 14 post-infection and were scanned using the μCT50. (A, B) Cortical bone loss (mm3) (A) and reactive bone formation (mm3) (B) from infected Il1r1+/+ and Il1r1-/- femurs were quantified using μCT analysis. Symbols represent individual data points from each mouse (Il1r1+/+ = circles, Il1r1-/- = squares), the top line of each bar represents the mean, and error bars represent standard deviation. Unpaired t-tests were used to compare cortical bone loss and reactive bone formation between Il1r1+/+ and Il1r1-/- mice. * p < 0.05, ** p < 0.01. (TIF) [file ppat.1007744.s006.tif]

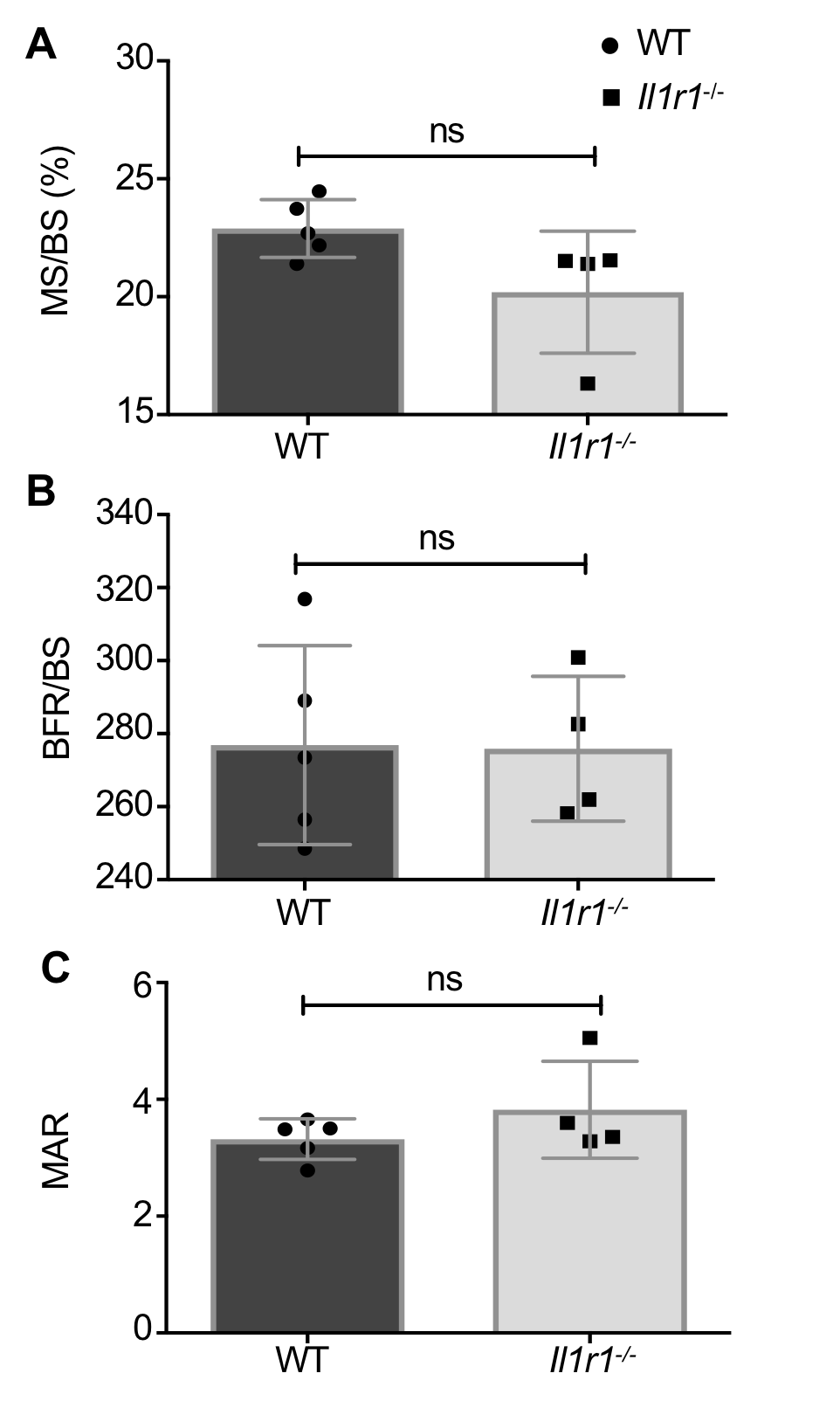

Supplement: S7 Fig — (A-C) Female mice were infected with 105 S. aureus CFUs (WT n = 5, Il1r1-/- n = 4), with 20 mg/kg calcein injected intraperitoneally on days 8 and 12 post-infection. Femurs were harvested at day 14 post-infection, and embedded in poly(methyl methacrylate) for sectioning. Calcein incorporated into the trabecular bone, and single- and double-labeled fluorescent surfaces were traced relative to total bone surface using OsteoMeasure software. (A-C) OsteoMeasure software was used to calculate mineralizing surface per bone surface (MS/BS) (%) (A), bone formation rate per bone surface (BFR/BS) (B), and mineral apposition rate (MAR) (C) from WT and Il1r1-/- infected femurs. Symbols represent individual data points from each mouse (WT = circles, Il1r1-/- = squares), the top line of each bar represents the mean, and error bars represent standard deviation. Unpaired t-tests were used to compare measurements of osteoblast activity in vivo between infected WT and Il1r1-/- mice. ns = not significant. (TIF) [file ppat.1007744.s007.tif]

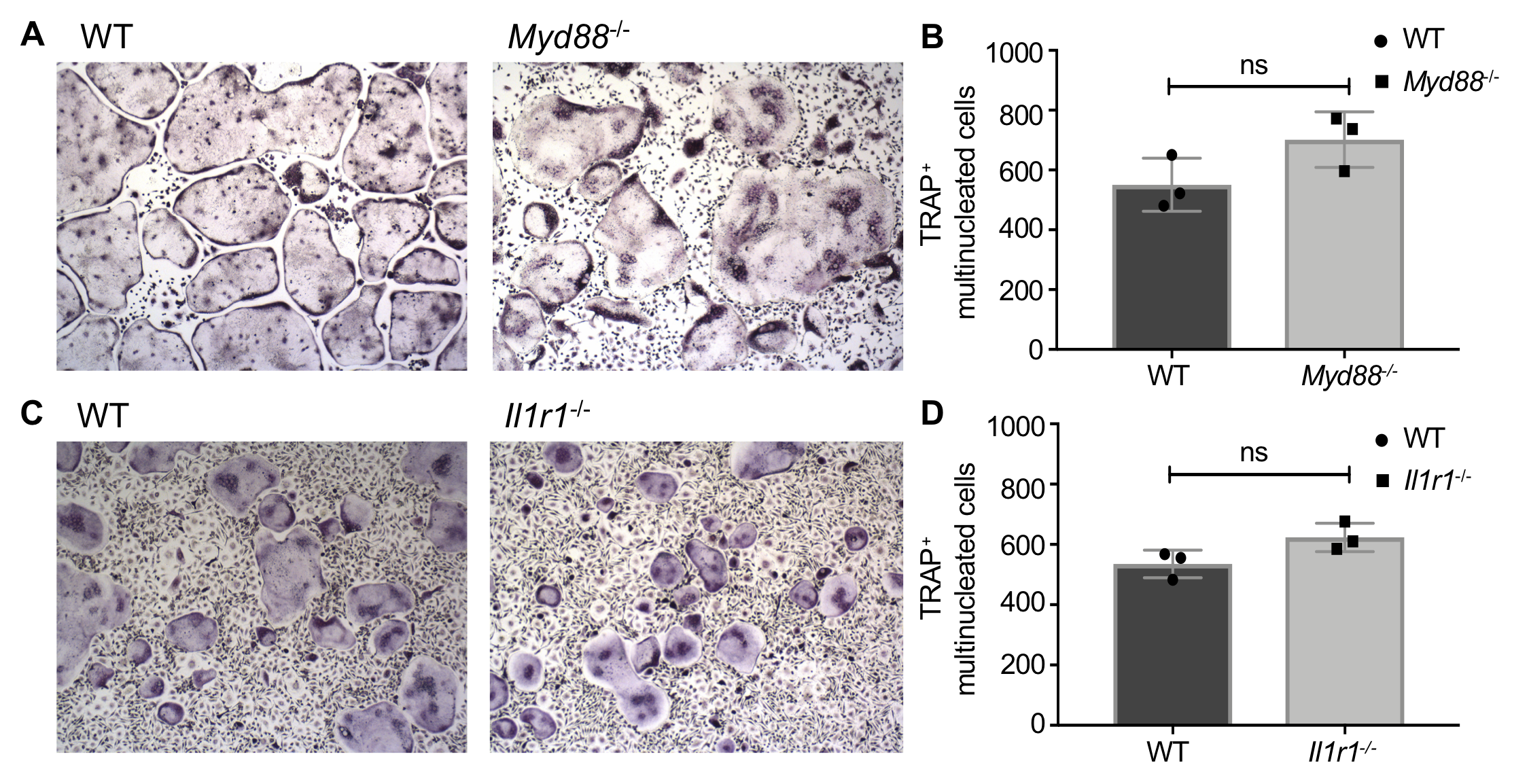

Supplement: S8 Fig — (A-D) WT, Myd88-/-, and Il1r1-/- BMMs were plated at 50,000 cells per well in a 96-well plate. Cell cultures were supplemented with 35 ng/mL RANKL and 1:20 CMG14-12 supernatant as an M-CSF source. Media and reagents were replenished on days 4 and 6 in culture (i.e. RANKL stimulation was continued for the entire experiment), and cells were fixed and stained for TRAP expression on day 7. (A-D) Cells were imaged at 10X (A, C) and TRAP+ multinucleated cells were counted using the OsteoMeasure software (B, D). Symbols represent individual well counts from (WT = circles, Myd88-/- and Il1r1-/- = squares), the top of each bar represents the mean, and error bars represent standard deviation. Unpaired t-tests were used to compare cell counts between WT and Myd88-/- or Il1r1-/- cells. ns = not significant. (TIF) [file ppat.1007744.s008.tif]
