# Supplementary material for: MyD88 and IL-1R signaling drive antibacterial immunity and osteoclast-driven bone loss during Staphylococcus aureus osteomyelitis
Source: PLoS Pathog. 2019 Apr 12;15(4):e1007744. doi: 10.1371/journal.ppat.1007744 (PMC6481883; doi:10.1371/journal.ppat.1007744)
Supplement: S1 Table — Femurs from female WT and Il1r1-/- mice were harvested at days 1, 3, 5, 10, and 14 post-infection with 105 S. aureus CFUs (n = 3 mice per timepoint). Femurs were homogenized in CelLytic Buffer MT and the supernatant from the bone homogenate was analyzed using a 32-plex Millipore kit on the Luminex platform to obtain cytokine abundance. The protein content in the femur lysate was quantified using a BCA kit. Cytokine data are reported as the mean pg cytokine/mg protein ± standard deviation, and were compared between infected WT and Il1r1-/- mice using multiple t-tests. * p < 0.05, ** p < 0.01, *** p < 0.001. (DOCX) [file ppat.1007744.s009.docx]

**S1 Table. Cytokine levels in WT and *Il1r1*^-/-^ mice during *S. aureus* osteomyelitis.**

|  | WT infected (white) | | | | |
| --- | --- | --- | --- | --- | --- |
|  | *Il1r1*^-/-^ infected (grey) | | | | |
|  | (pg cytokine/mg protein) | | | | |
| Cytokine | Day 1 | Day 3 | Day 5 | Day 10 | Day 14 |
| IL-1α | 217.7 ± 60.2 | 226.9 ± 82.2 | 212.5 ± 242.3 | 544.1 ± 545.0 | 241.2 ± 76.6 |
|  | 238.5 ± 40.2 | 386.6 ± 73.3 | 432.8 ± 243.2 | 693.3 ± 50.4 | 965.3 ± 826.0 |
| IL-1β | 500.1 ± 105.2* | 408.5 ± 114.3 | 188.8 ± 146.7 | 199.9 ± 150.8 | 116.4 ± 38.5 |
|  | 855.2 ± 93.8 | 402.5 ± 147.1 | 802.8 ± 471.2 | 314.2 ± 132.8 | 457.1 ± 305.2 |
| IL-2 | 4.0 ± 0.5 | 1.4 ± 0.2 | 1.0 ± 0.2* | 2.1 ± 0.6 | 2.0 ± 0.4 |
|  | 4.6 ± 0.9 | 1.1 ± 0.1 | 1.7 ± 0.2 | 1.8 ± 0.1 | 2.1 ± 0.8 |
| IL-3 | 0.5 ± 0.1 | 1.1 ± 0.2 | 1.3 ± 0.2 | 1.5 ± 0.5 | 1.5 ± 0.2 |
|  | 0.7 ± 0.2 | 1.1 ± 0.4 | 2.5 ± 1.5 | 1.0 ± 0.1 | 1.3 ± 0.4 |
| IL-4 | 2.4 ± 0.1 | 1.6 ± 0.1 | 1.3 ± 0.1* | 2.4 ± 1.1 | 10.9 ± 6.5 |
|  | 2.1 ± 0.2 | 1.8 ± 0.2 | 2.3 ± 0.5 | 2.8 ± 0.9 | 15.7 ± 18.2 |
| IL-5 | 6.2 ± 1.8 | 2.3 ± 1.4 | 1.7 ± 1.8 | 3.3 ± 2.4 | 2.8 ± 1.6 |
|  | 7.0 ± 3.4 | 3.7 ± 2.6 | 5.4 ± 3.2 | 5.2 ± 0.3 | 7.0 ± 3.6 |
| IL-6 | 724.6 ± 227.9 | 151.0 ± 50.6 | 51.8 ± 4.6** | 144.1 ± 114.8 | 68.1 ± 30.9 |
|  | 502.1 ± 136.3 | 86.4 ± 17.7 | 173.9 ± 43.9 | 69.0 ± 13.4 | 118.1 ± 131.9 |
| IL-7 | 3.1 ± 0.4 | 2.2 ± 0.3 | 2.1 ± 0.4* | 3.0 ± 0.4 | 3.3 ± 0.3 |
|  | 2.8 ± 0.6 | 2.2 ± 0.5 | 2.7 ± 0.2 | 3.0 ± 0.1 | 2.6 ± 0.7 |
| IL-9 | 208.3 ± 18.8 | 116.3 ± 7.9 | 112.4 ± 10.5 | 153.0 ± 26.0 | 129.2 ± 25.2 |
|  | 225.1 ± 41.3 | 103 ± 15.5 | 114.8 ± 15.9 | 133.2 ± 5.1 | 131.5 ± 20.9 |
| IL-10 | 5.1 ± 0.7 | 5.4 ± 0.4 | 5.1 ± 0.5* | 6.4 ± 2.4 | 7.2 ± 1.0 |
|  | 5.6 ± 0.5 | 5.1 ± 1.1 | 8.4 ± 1.3 | 6.8 ± 0.8 | 10.0 ± 5.1 |
| IL-12 p40 | 0.8 ± 0.0 | 17.9 ± 6.9 | 27.9 ± 6.4 | 21.1 ± 4.3 | 46.5 ± 22.9 |
|  | 0.7 ± 0.1 | 20.4 ± 12.0 | 34.3 ± 13.5 | 18.9 ± 6.0 | 24.2 ± 9.5 |
| IL-12 p70 | 9.8 ± 3.0 | 6.8 ± 1.1 | 8.3 ± 1.7 | 8.6 ± 4.4 | 8.2 ± 0.9 |
|  | 6.3 ± 0.6 | 6.6 ± 2.2 | 7.9 ± 0.7 | 4.2 ± 1.1 | 6.1 ± 2.9 |
| IL-15 | 16.9 ± 3.8 | 13.3 ± 4.3 | 9.0 ± 1.8** | 14.8 ± 6.8 | 12.4 ± 1.0* |
|  | 16.1 ± 5.2 | 10.8 ± 2.8 | 17.5 ± 0.6 | 14.1 ± 1.1 | 12.3 ± 7.3 |
| IL-17 | 4.6 ± 0.5 | 18.0 ± 6.4* | 14.9 ± 10.9 | 233.5 ± 208.3 | 206.8 ± 43.2** |
|  | 4.5 ± 0.6 | 3.3 ± 1.5 | 29.5 ± 36.4 | 6.2 ± 2.4 | 9.3 ± 5.6 |
| IFNy | 10.1 ± 1.7 | 6.3 ± 3.3 | 5.2 ± 2.0** | 12.0 ± 8.2 | 12.0 ± 4.8 |
|  | 10.0 ± 1.8 | 8.2 ± 2.1 | 20.1 ± 2.6 | 17.4 ± 5.2 | 16.8 ± 3.0 |
| LIF | 133.4 ± 11.5 | 99.3 ± 9.0 | 84.5 ± 31.8 | 111.2 ± 38.3 | 98.9 ± 12.2 |
|  | 157.3 ± 28.1 | 92.5 ± 13.0 | 112.5 ± 24.1 | 111.7 ± 12.0 | 130.6 ± 32.0 |
| TNFα | 40.2 ± 5.7 | 56.4 ± 10.7 | 38.7 ± 16.5 | 64.9 ± 38.6 | 52.3 ± 5.2 |
|  | 29.8 ± 5.1 | 53.2 ± 8.9 | 71.6 ± 24.7 | 60.5 ± 14.7 | 80.3 ± 37.1 |
| G-CSF | 6012.0 ± 1879.3* | 624.0 ± 215.4 | 359.6 ± 165.8 | 809.4 ± 495.4 | 555.6 ± 161.3 |
|  | 1928.9 ± 84.7 | 678.3 ± 120.2 | 1115.0 ± 454.3 | 1285.6 ± 504.4 | 2339.3 ± 1701.6 |

|  | WT infected (white) | | | | |
| --- | --- | --- | --- | --- | --- |
|  | *Il1r1*^-/-^ infected (grey) | | | | |
|  | (pg cytokine/mg protein) | | | | |
| Cytokine | Day 1 | Day 3 | Day 5 | Day 10 | Day 14 |
| GM-CSF | 37.3 ± 6.6* | 10.2 ± 6.4 | 3.3 ± 2.9** | 13.3 ± 10.0 | 5.2 ± 4.1 |
|  | 25.1 ± 2.1 | 6.6 ± 1.7 | 13.7 ± 2.6 | 7.6 ± 2.4 | 10.8 ± 10.0 |
| M-CSF | 1898.9 ± 490.3 | 807.7 ± 651.3 | 761.0 ± 1010.1 | 92.2 ± 53.4 | 89.2 ± 27.4 |
|  | 936.5 ± 354.7 | 702.6 ± 128.9 | 1452.5 ± 1163.2 | 119.0 ± 75.2 | 316.8 ± 344.4 |
| VEGF | 184.0 ± 26.9 | 360.1 ± 74.2 | 358.9 ± 56.7 | 365. 0 ± 169.5 | 338.2 ± 201.8 |
|  | 187.4 ± 52.9 | 281.4 ± 103.4 | 306.3 ± 74.8 | 237.4 ± 58.5 | 179.1 ± 115.6 |
| CCL2 / MCP-1 | 343.8 ± 95.7 | 178.8 ± 136.0 | 122.3 ± 126.5 | 63.6 ± 30.3 | 66.1 ± 2.2 |
|  | 200.9 ± 36.4 | 113.0 ± 20.6 | 372.0 ± 144.2 | 73.2 ± 12.2 | 86.6 ± 62.1 |
| CCL3 / MIP-1α | 233.2 ± 42.0 | 258.6 ± 62.1 | 181.7 ± 82.5 | 319.3 ± 216.5 | 297.9 ± 5.04 |
|  | 324.7 ± 75.3 | 332.5 ± 63.9 | 452.4 ± 177.2 | 487.1 ± 145.7 | 750.1 ± 421.1 |
| CCL4 / MIP-1β | 274.4 ± 70.1 | 181.2 ± 46.7 | 131.1 ± 66.0 | 305.0 ± 235.6 | 366.8 ± 90.3 |
|  | 472.8 ± 126.5 | 274.2 ± 41.4 | 364.1 ± 168.6 | 410.9 ± 131.5 | 760.6 ± 441.9 |
| CCL5 / RANTES | 20.9 ± 3.1 | 25.2 ± 2.5* | 42.2 ± 9.5 | 106.8 ± 63.2 | 89.8 ± 16.7 |
|  | 22.1 ± 3.2 | 50.7 ± 13.8 | 64.5 ± 14.8 | 92.4 ± 30.8 | 97.9 ± 14.4 |
| CCL11 / Eotaxin | 178.6 ± 9.3 | 368.9 ± 35.9 | 289.3 ± 29.9* | 421.9 ± 63.6 | 516.7 ± 61.4* |
|  | 164.2 ± 24.5 | 342.3 ± 35.1 | 395.8 ± 55.2 | 364.1 ± 33.4 | 322.3 ± 53.4 |
| CXCL1 / KC | 1619.2 ± 447.1 | 532.5 ± 178.8 | 281.7 ± 89.1*** | 602.0 ± 378.9 | 385.5 ± 165.4 |
|  | 1029.8 ± 165.2 | 353.5 ± 27.3 | 809.6 ± 54.5 | 412.4 ± 156.7 | 541.3 ± 2453 |
| CXCL2 / MIP-2 | 5713.9 ± 1425.8 | 7811.6 ± 3065.5 | 5648.5 ± 3960.1 | 6162.9 ± 4560.5 | 1977.1 ± 1209.6* |
|  | 6414.7 ± 918.3 | 7358.6 ± 2161.2 | 12454.7 ± 2926.9 | 12014.6 ± 569.7 | 10345.3 ± 4458.9 |
| CXCL5 / LIX | 364.3 ± 116.3 | 552.4 ± 241.3 | 399.8 ± 346.4 | 475.7 ± 290.3 | 336.9 ± 172.4 |
|  | 393.0 ± 36.7 | 522.6 ± 113.9 | 873.3 ± 227.8 | 712.1 ± 262.4 | 683.0 ± 273.0 |
| CXCL9 / MIG | 121.7 ± 91.8 | 162.0 ± 24.4** | 152.2 ± 37.2* | 763.8 ± 596.5 | 712.7 ± 29.8 |
|  | 92.1 ± 14.1 | 352.1 ± 60.6 | 673.1 ± 229.8 | 1301.7 ± 342.1 | 1042.2 ± 375.8 |
| CXCL10 / IP-10 | 394.3 ± 369.1 | 290.8 ± 57.4 | 170.6 ± 42.0*** | 363.4 ± 151.8 | 312.1 ± 70.9 |
|  | 214.8 ± 26.4 | 399.8 ± 42.8 | 594.5 ± 62.7 | 534.1 ± 78.2 | 412.8 ± 87.9 |
